# Bulk and Surface Contributions to Ionisation Potentials of Metal Oxides

**DOI:** 10.1002/anie.202308411

**Published:** 2023-08-24

**Authors:** Xingfan Zhang, Taifeng Liu, Lei Zhu, Jingcheng Guan, You Lu, Thomas W. Keal, John Buckeridge, C. Richard A. Catlow, Alexey A. Sokol

**Affiliations:** ^1^ Kathleen Lonsdale Materials Chemistry Department of Chemistry University College London WC1H 0AJ London UK; ^2^ Scientific Computing Department STFC Daresbury Laboratory WA4 4AD Warrington Cheshire UK; ^3^ School of Engineering London South Bank University SE1 OAA London UK; ^4^ School of Chemistry Cardiff University Park Place CF10 1AT Cardiff UK; ^5^ National & Local Joint Engineering Research Center for Applied Technology of Hybrid Nanomaterials Henan University 475004 Kaifeng China

**Keywords:** Ceria, Ionization Potential, Metal Oxides, QM/MM, Surface Chemistry

## Abstract

Determining the absolute band edge positions in solid materials is crucial for optimising their performance in wide‐ranging applications including photocatalysis and electronic devices. However, obtaining absolute energies is challenging, as seen in CeO_2_, where experimental measurements show substantial discrepancies in the ionisation potential (IP). Here, we have combined several theoretical approaches, from classical electrostatics to quantum mechanics, to elucidate the bulk and surface contributions to the IP of metal oxides. We have determined a theoretical bulk contribution to the IP of stoichiometric CeO_2_ of only 5.38 eV, while surface orientation results in intrinsic IP variations ranging from 4.2 eV to 8.2 eV. Highly tuneable IPs were also found in TiO_2_, ZrO_2_, and HfO_2_, in which surface polarisation plays a pivotal role in long‐range energy level shifting. Our analysis, in addition to rationalising the observed range of experimental results, provides a firm basis for future interpretations of experimental and computational studies of oxide band structures.

## Graphical abstract

Using a combination of theoretical techniques, we separated the bulk and surface contributions of ionisation potentials of metal oxides, highlighting the critical role of electrostatics. Low bulk ionisation potentials and a high degree of tunability from surfaces were found in CeO_2_, HfO_2_, and ZrO_2_, explaining the discrepancies from measurements and guiding the future design of band‐edge‐related photocatalytic, photovoltaic, and electrochemical devices.

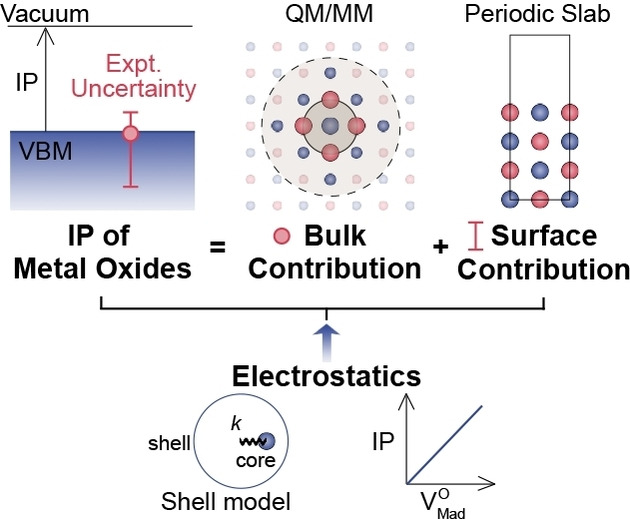

## Introduction

The ionisation potential (IP), electron affinity (EA), and work function (*Φ*) are fundamental quantities of metal oxides governing electronic, optical and transport processes. While isolated molecules have well‐defined IPs and EAs that can be accurately measured or calculated using experimental and computational techniques,^[1]^ obtaining the absolute band‐edge positions in solids with respect to the vacuum level is far more challenging. In the solid state, these quantities become surface‐related parameters, and experimental measurements on the same material can exhibit significant variations due to different morphologies and processing histories.^[2]^

Compared to the highly variable nature of *Φ*,^[3]^ the surface sensitivity of IP in metal oxides remains less clearly understood. An intriguing case is provided by ceria (CeO_2_), which has a wide range of applications in heterogeneous catalysis.^[4]^ Recently, Wardenga and Klein^[5]^ reported IPs of undoped CeO_2_ thin films prepared using radio frequency magnetron sputtering. Surprisingly, the measured IPs on different samples varied from 6.5 to 9.1 eV, which is a substantial scatter compared with other oxides prepared with similar techniques.[^3^, ^6^] Significant variations (from 5.47 eV to 7.7 eV)^[7]^ are also observed in measurements on other CeO_2_ samples (Table S1). A possible origin for the IP variation in CeO_2_ is its highly variable surface chemistry,^[5]^ which can release and store oxygen repeatedly in redox cycles.^[8]^ However, such a significant variation is not seen in other easily reducible oxides, indicating that some other previously unaccounted for factors play a significant role.

Here, we combine several computational approaches: classical electrostatic analyses, hybrid quantum mechanics/molecular mechanics (QM/MM) embedded‐cluster simulations,^[9]^ and plane‐wave density functional theory (DFT) calculations,^[10]^ to disentangle the origins of the highly variable IP of CeO_2_. We separate the bulk and several sources of surface contributions to the IP using theoretical models, elucidating another cause of uncertainty in experimental measurements apart from the variable surface chemistry. We find critical intrinsic surface effects in metal oxides, particularly prominent in CeO_2_ and other high‐dielectric‐constant (high‐*κ*) MO_2_ oxides, where surface polarisation strongly affects the absolute band positions deep into the bulk. Our approach enables a clear and coherent approach to computational and experimental studies of this crucially important quantity for both ceria and other oxides.

## Results and Discussion

### Surface polarisation mechanisms of metal oxides and effects on ionisation potential

**Figure 1:**
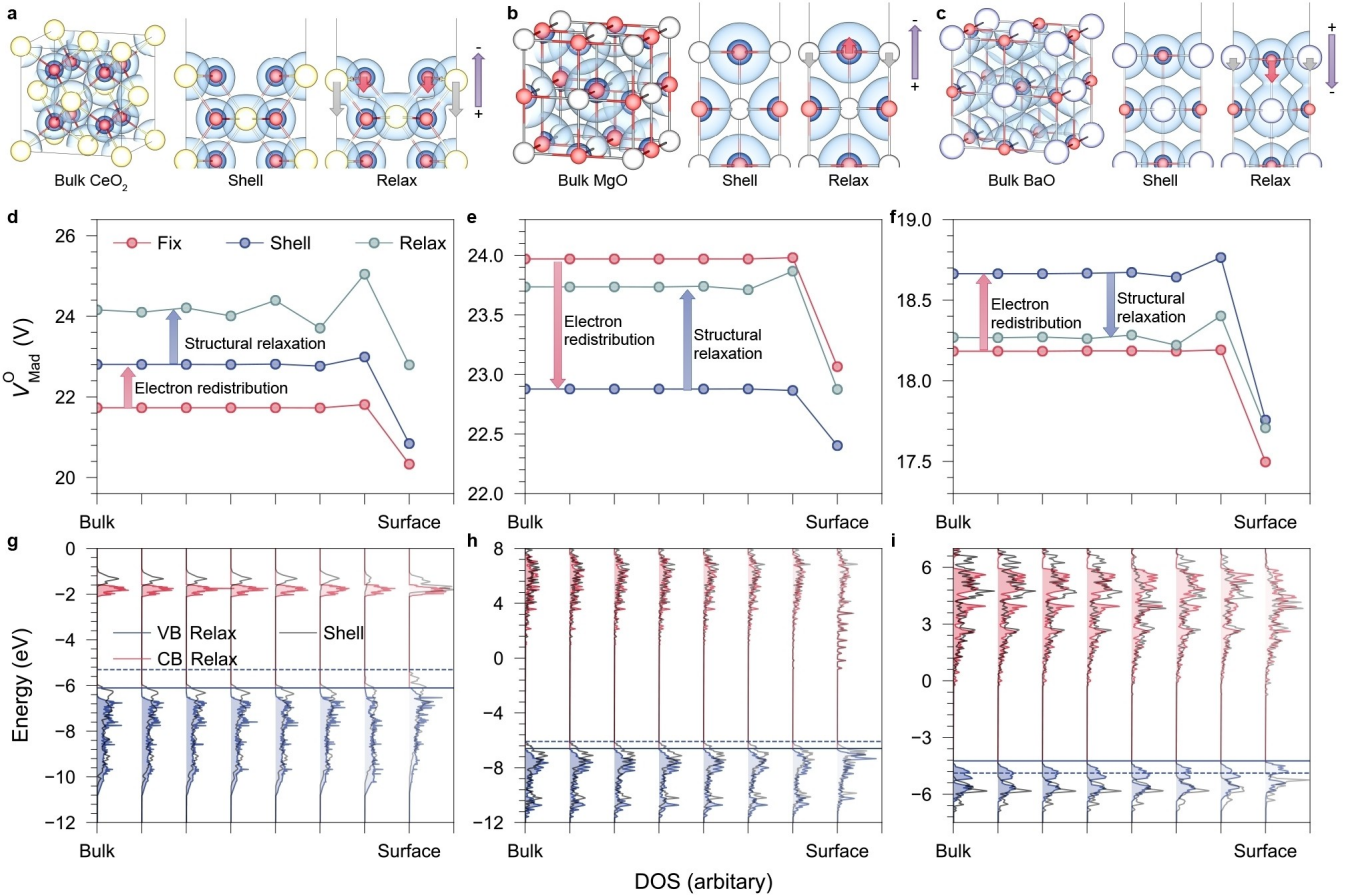
Effects of surface polarisation and structural relaxation on the ionisation potential: the case of the nonpolar CeO_2_(110), MgO(100), and BaO(100) surfaces. (a–c) Charge density in bulk calculated within the 3D periodicity, on nonpolar surfaces with fixed bulk geometry (denoted as “Shell”), and after structural relaxation (“Relax”). Red, yellow, white, and purple spheres indicate O, Ce, Mg, and Ba, respectively. The dark isosurface represents a level of 0.5 *e*/Bohr^3^, while the light blue isosurface is 0.08 *e*/Bohr^3^ for CeO_2_ and 0.03 *e*/Bohr^3^ for MgO and BaO, respectively. Arrows indicate the relative displacements of surface ions after the structural relaxation, resulting in different types of surface rumpling, which suppress the ionisation on CeO_2_(110) and MgO(100) but promote the ionisation on BaO(100) due to the opposite directions of near‐surface electrostatic fields. (d–f) The O‐site electrostatic (Madelung) potentials from the bulk region to the surface, modelled by the shell model, showing the variation of the electrostatic environment due to initial electronic polarisation (from “Fix” to “Shell”) and further structural relaxation (from “Shell” to “Relax”). (g–i) The layer‐by‐layer density of states from bulk to surface confirms the shifts of the valence band edges before (dash lines) and after structural relaxation (solid lines) due to different mechanisms of surface rumpling. The intensities of the valence band of CeO_2_, the conduction bands of MgO and BaO have been magnified threefold for better clarity.

We first compare cubic, fluorite structured CeO_2_ with two rock‐salt structured oxides, MgO and BaO, to understand how cationic properties affect the bonding environment and surface polarisation. First, periodic DFT calculations using the Vienna Ab‐initio Simulation Package (VASP)^[10]^ at the PBE0^[11]^ level of theory were performed to compare charge densities in bulk and at their nonpolar surfaces (Figure 1a–c). In MgO, O^2−^ is much more diffuse than Mg^2+^, and Mg^2+^ has a low polarisability, which we calculate as 0.48 a.u. in the gas phase. By contrast, Ce^4+^ has an over tenfold higher calculated polarisability of 5.87 a.u., in the gas phase associated with the more diffuse charge density of Ce^4+^. Ba^2+^ has an even higher calculated polarisability of 10.42 a.u. and a large Shannon ionic radius,^[12]^ and the cation charge density in BaO is more diffuse than that of the anions.

To distinguish and quantify various sources of surface effects on energy‐level shifting, we combined DFT calculations with electrostatic analyses supported by shell‐model (SM) interatomic potentials^[13]^ as implemented in the General Utility Lattice Package (GULP),^[14]^ using the nonpolar (Tasker Type 1)^[15]^ CeO_2_(110), MgO(100), and BaO(100) surfaces as typical examples.

In the SM, an ion is separated into a massless shell and an atomic core connected by a harmonic spring, parameterised to reproduce the ionic polarisability and hence dielectric constants. Here, we denote “Shell” as the frozen‐nuclei surface slab model retaining the bulk geometry, where only electronic degrees of freedom are relaxed, representing the polarised electron structure due to surface termination. This state can be obtained from either a DFT single‐point calculation or shell relaxation using the SM. “Relax” represents the optimised geometry that further considers atomic core relaxation. Finally, the SM allows the investigation of the just‐cleaved unpolarised state without electronic redistribution, in which the shells are fixed at the core sites as those in the bulk environment, thereby preventing the surface polarisation (denoted as “Fix”).

When a nonpolar surface of an ionic crystal is cleaved from the bulk, the net dipole moment is zero due to the complete cancellation of ionic charges in each atomic plane. Consequently, the nonpolar arrangement of atomic layers does not affect the electrostatic potentials on ions in the bulk at the “Fix” state, making it an ideal model for studying the contributions of electronic redistribution and atomic relaxation on surface‐induced energy‐level shifting. Although CeO_2_(110) is not the most stable surface of ceria, it can be controllably synthesised in experiments, leading to enhanced activity in some particular catalytic reactions such as CO oxidation.^[16]^

For the “Shell” state, DFT calculations on CeO_2_(110) show deformed charge density on both surface Ce^4+^ and O^2−^ sites compared with the symmetrical distribution in the bulk (Figure 1a). The charge density on MgO(100) is also polarised but exhibits a lesser deformation. Figure 1d–f shows the variation of the calculated Madelung potential^[17]^ on oxygen core sites ($V_{Mad}^{O}$ ) from bulk to surface using the SM. The vacuum levels have been aligned to 0 V for a direct comparison of the absolute $V_{Mad}^{O}$ in various systems. In metal oxides, the valence band maximum (VBM) is dominated by the O 2*p* orbitals, and the IP is determined mainly by the balance between the local electrostatic environment (represented by $V_{Mad}^{O}$ ) and the second electron affinity of oxygen ($A_{O}^{2}$ ).^[2b]^ $A_{O}^{2}$ is a lattice‐sensitive variable,^[18]^ but a higher $V_{Mad}^{O}$ indicates stronger electron binding to the atomic site and a higher IP. For CeO_2_ and BaO, the electronic redistribution (from “Fix” to “Shell”) on their respective nonpolar surfaces increases $V_{Mad}^{O}$ on all anion sites across the slab; in contrast, $V_{Mad}^{O}$ under MgO(100) decreases after the electronic redistribution.

The shift of $V_{Mad}^{O}$ in the middle of the slab (*D*_s_) from the “Fix” to “Shell” states has been used to evaluate the magnitude of surface polarisation, which serves as an appropriate correction to the DFT‐based “core‐level alignment” (CLA) method to evaluate the bulk IP (details in Supporting Information).^[19]^ For MgO(100), surface polarisation contributes +0.85 eV to the IP, yielding 6.89 eV after the *D*_s_ correction at the PBE0 level of theory, consistent with the experimental measurement of 7.15 eV.^[20]^ For CeO_2_, the uncorrected CLA approach overestimates the bulk IP by 1.06 eV due to the opposite multipolar shift, resulting in 4.76 eV after the *D*_s_ correction. Similarly, a negative *D*_s_ correction (−0.61 eV) is observed in BaO, whose cation polarisability is also much higher than that of Mg^2+^. The group 2 rock‐salt structured oxides share a similar in‐lattice anion polarisability from 6.3 to 7.0 a.u. as calculated by an embedded‐cluster model (Table S17), while cation polarisabilities vary substantially. For example, Ca^2+^, with a calculated polarisability of 3.27 a.u., has a positive but much lower *D*_s_ shift in CaO (+0.17 eV), compared to Mg^2+^ in MgO. Sr^2+^ is more polarisable (calculated as 5.82 a.u.) and close to the in‐lattice polarisability of O^2−^ in SrO, making the *D*_s_ shift (−0.06 eV) small. The impact of the cation‐anion relative polarisability on surface polarisation can be understood by a single‐layer atomistic model (Figure S1a). We calculated the shell displacement in response to a constant electric field with variable cation polarisability and lattice constant, for simulating the rock‐salt structure (100) surface polarisation (Figure S1b). As the cation polarisability increases, the shell displacement on cations becomes more pronounced and gradually exceeds that on anions. Hence, on descending the group 2 oxides, cations play an increasing role in surface polarisation, transferring the *D*_s_ shift from positive to negative, and shifting the VBM accordingly in opposite directions.

### Effects of surface rumpling

The variation of $V_{Mad}^{O}$ from “Shell” to “Relax” as seen in Figure 1d–f reveals the influence of structural relaxation on the electrostatic environment. Surface rumpling occurs on relaxation, i.e., surface ions are displaced by different amounts. On CeO_2_(110), cations move further inwards than anions. On MgO(100), while Mg^2+^ moves inwards, O^2−^ protrudes beyond the original plane. The relaxed surfaces are both terminated by oxygen, consistent with previous theoretical and experimental studies.^[21]^ Structural relaxation results in an electrostatic field normal to the surface due to charge separation (Figure 1a–b). The electrostatic fields on MgO(100) and CeO_2_(110) suppress the electron transfer into the vacuum, shown by the positive shift of $V_{Mad}^{O}$ from “Shell” to “Relax” in Figure 1d–e. However, surface rumpling on BaO(100) differs significantly: as anions move further towards the bulk than cations, an electrostatic field opposite to those on MgO(100) and CeO_2_(110) is generated by the Ba‐terminated topmost plane. This surface rumpling mechanism promotes the ionisation of electrons on BaO(100), in agreement with the negative shift of $V_{Mad}^{O}$ (Figure 1f). In Figure 1g–i, we present the layer‐by‐layer density of states before (dash lines) and after (solid lines) structural relaxation obtained by DFT calculations on slab models. Electronic‐structure calculations confirm the shift of VBM from −5.31 eV to −6.11 eV for CeO_2_(110) and from −6.08 eV to −6.60 eV for MgO(100) due to oxygen‐terminated surface rumpling, while the protruded cations on BaO(100) shift the VBM in the opposite direction from −4.88 eV to −4.24 eV. Our theoretical calculations consistently illustrate both the differences and similarities in surface polarisation and structural relaxation mechanisms among the three oxides, highlighting the intricate relationship between surface structures and energy‐level shifting.

### Long‐range surface effects on the bulk electrostatic environment

**Figure 2:**
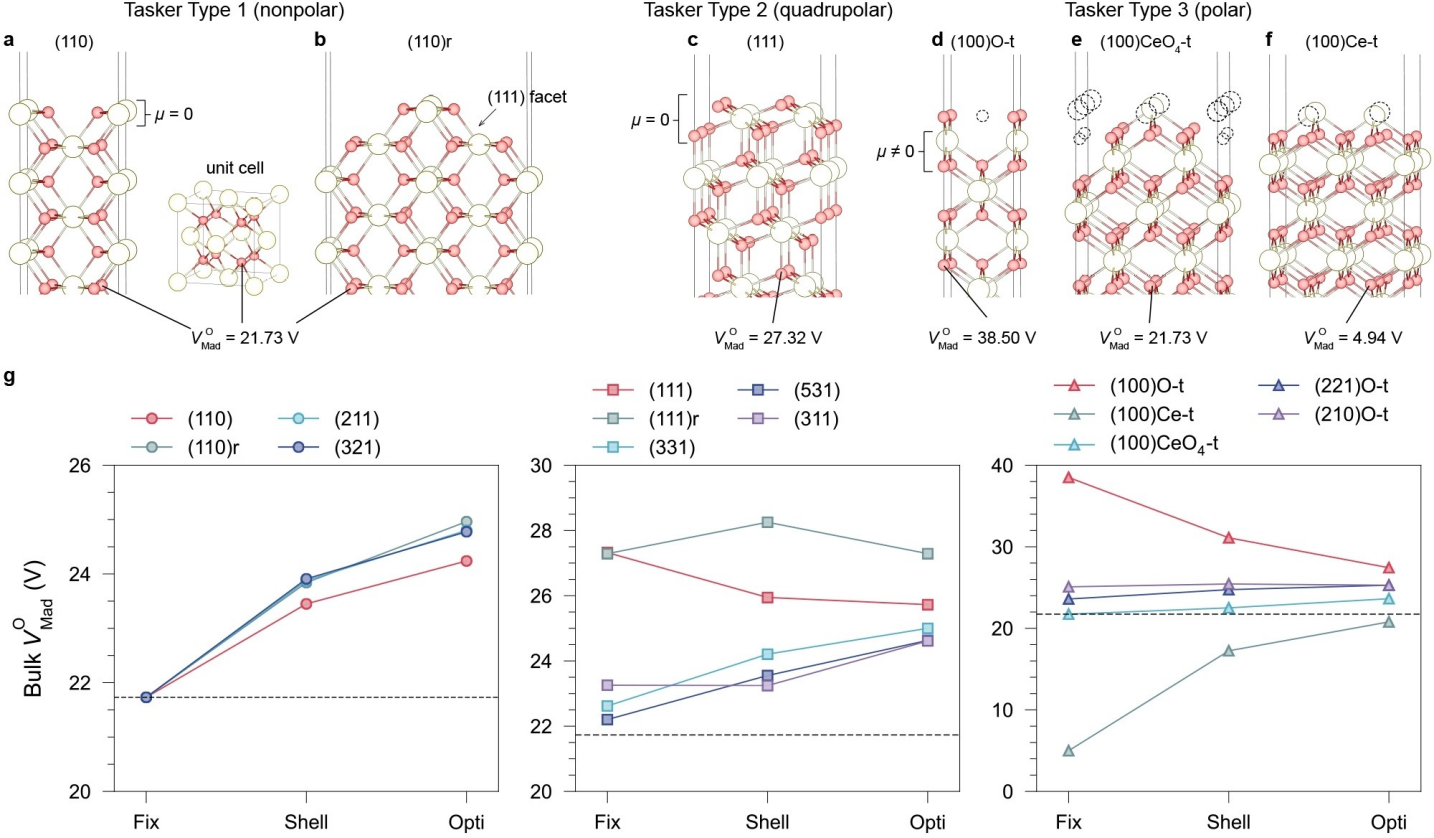
Long‐range surface termination effects on the bulk electrostatic potentials in CeO_2_. The results are categorised as polar, nonpolar, and quadrupolar surfaces as defined by Tasker.^[15]^ Slab models of (a) the nonpolar (110) surface and (b) its (2×1) reconstruction, (c) the quadrupolar (111) surface, and (d–f) three different reconstruction patterns of the polar (100) surface with fixed bulk geometry. The dashed circles represent the atomic vacancies due to surface reconstruction. The $V_{Mad}^{O}$ values shown below indicate the converged Madelung potential on O sites in the middle of slabs at the “Fix” state, representing the bulk electrostatic environment. (g) Variation of the bulk $V_{Mad}^{O}$ under different surface terminations after electronic redistribution (the “Shell” state) and structural relaxation (the “Relax” state). Dash lines represent the $V_{Mad}^{O}$ calculated under 3D periodicity that excludes surface effects (21.73 V). The deviations of $V_{Mad}^{O}$ from 21.73 V indicate the effect of long‐range surface polarisation on varying the bulk electrostatic environment.

Oxides can have both quadrupolar and polar as well as the nonpolar surfaces discussed above. The catalytic performance of CeO_2_ is known to be highly dependent on the crystal orientation.[^16a^, ^22^] For the nonpolar surfaces, in the “Fix” state, each atomic plane is charge neutral, ensuring that the Madelung potentials in deeper planes (bulk) are consistent with those calculated in the three‐dimensional (3D) periodic cell, i.e., no long‐range polarisation, as seen in CeO_2_(110) (Figure 2a). However, quadrupolar (Tasker Type 2) and polar surfaces (Tasker Type 3) have entirely different stacking sequences. Although the net dipole moment remains zero through counterbalanced periodic charges on quadrupolar surfaces (Figure 2c) and appropriate reconstructions^[23]^ on polar surfaces (Figure 2d–f), the intrinsic higher multipoles along the normal direction of the surface also affect the electrostatic potential. Figure 2g shows the calculated bulk $V_{Mad}^{O}$ in CeO_2_ under different surface terminations at the “Fix” state, demonstrating that the stacking sequence affects the $V_{Mad}^{O}$ deep into the bulk. Typically, the bulk $V_{Mad}^{O}$ is elevated by O‐terminated surfaces, decreased by Ce‐terminated surfaces, and remains unchanged under “fixed” nonpolar terminations. Electronic redistribution and atomic relaxation further affect the bulk potential and usually result in increased $V_{Mad}^{O}$ compared with the ideal 21.73 V, which excludes surface effects.

From previous electrostatic analyses, we conclude that surface effects on the energy‐level shifting in metal oxides can be separated into long‐range and short‐range components. On the one hand, the electrostatic potential varies from the surface to the bulk due to differences in the atomic bonding environment, resulting in short‐range band bending near the surface. On the other, long‐range surface polarisation affects the bulk energy levels, acting as a voltage that constantly shifts the average electrostatic potential across the slab. In realistic materials with a finite size, the IP and EA measured by experimental techniques are always influenced by surface terminations. Even when measurements are from the deep layers of samples, the energy levels are readily shifted due to long‐range surface polarisation. The experimentally measured “bulk” result is therefore a different quantity from the theoretical bulk IP, which, by definition, excludes all surface effects.

### Determination of the bulk ionisation potential

**Figure 3:**
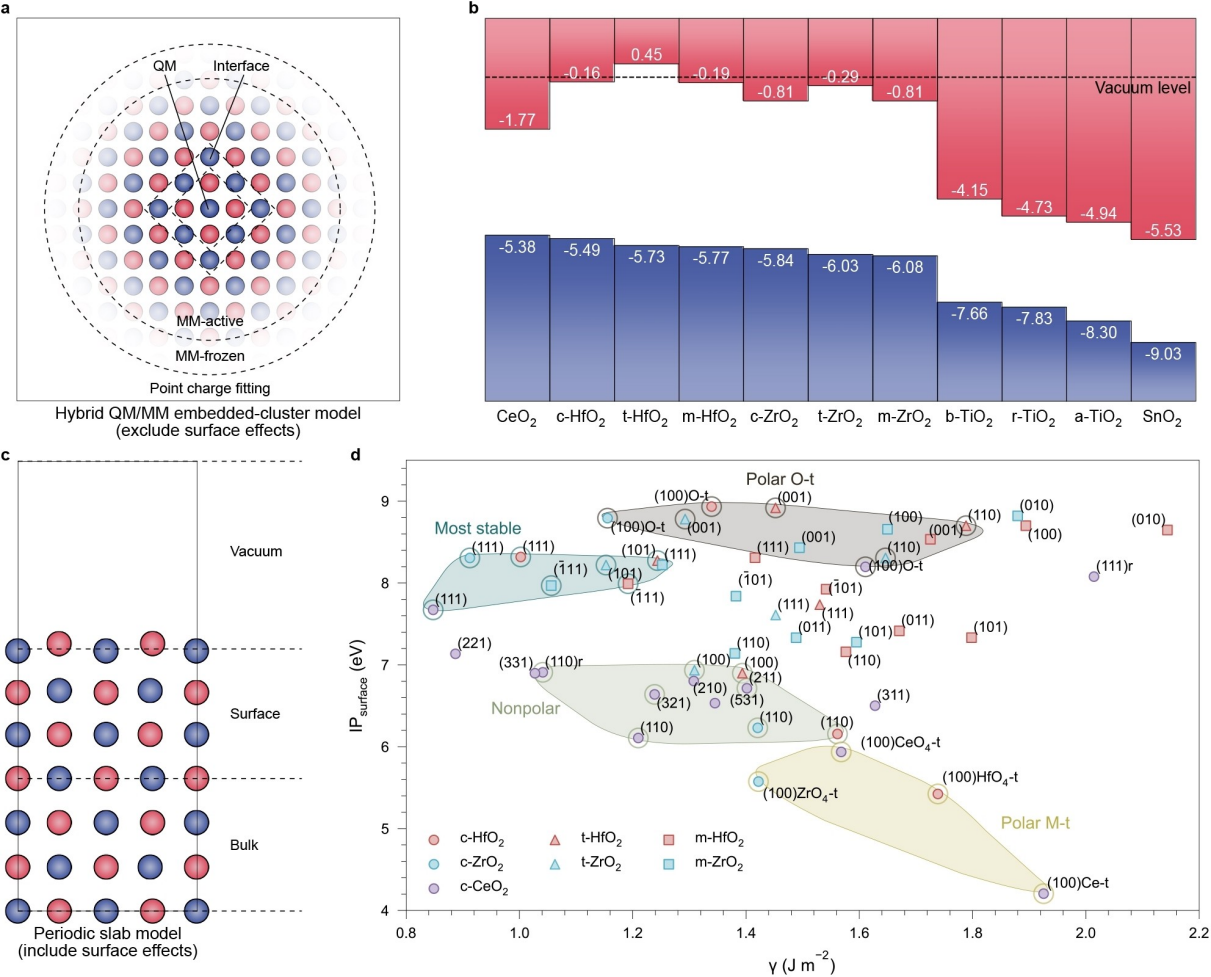
Theoretical approaches for determining the bulk and surface contributions to the ionisation potential of solids and their applications to MO_2_ oxides. (a) Hybrid QM/MM embedded‐cluster approach that excludes all surface effects for calculating the bulk contribution to the IP. (b) Intrinsic band alignment of MO_2_‐type oxides with respect to a common reference vacuum level. The valence band alignments are determined by the QM/MM approach at the BB1K level of theory, while positions of conduction band minima (CBM) are determined by adding the theoretical band gaps calculated by the plane‐wave DFT approach with the HSE06 functional. Previous results on TiO_2_ polymorphs^[25]^ and SnO_2_ ^[30]^ are also included. (c) Periodic slab model to calculate the surface‐dependent IP. (d) Surface energies and IPs of pristine surfaces of CeO_2_, ZrO_2_ and HfO_2_. Four regions are highlighted in different colours to distinguish the O‐terminated polar surfaces, the most stable (quadrupolar) surface in each phase, nonpolar surfaces, and metal‐terminated polar surfaces. The scatter points without circle frames are not included in these regions.

The bulk IP is a quantity reflecting the intrinsic electronic structure of a material and is independent of surface terminations. The $V_{Mad}^{O}$ calculated within 3D periodicity is such a quantity that indicates the intrinsic electrostatic environment inside a material and determines the band edge positions.^[24]^ For more accurate electronic‐structure calculations, the key to obtaining reliable bulk IPs is to exclude surface effects using appropriate models. The hybrid QM/MM embedded‐cluster model (Figure 3a) is the state‐of‐the‐art approach to determining the bulk IP, which reproduces the bulk electrostatic environment in the MM regions, without surface effects, while maintaining access to the vacuum level.[^9^, ^25^] Our QM/MM calculations run with the ChemShell code^[26]^ predict bulk IPs of 5.38 eV, 5.10 eV, 4.99 eV, and 4.92 eV for CeO_2_ using the BB1K,^[27]^ PBE0,^[11]^ HSE06,^[28]^ and B97‐2^[29]^ hybrid functionals, respectively. The predictions show a slightly increasing trend with the percentage of Hartree‐Fock exchange in hybrid functionals, which is also observed in other oxides (Table S15).^[30]^ The screening effect considered by HSE06 leads to a slightly lower calculated IP compared to PBE0 with an equivalent amount of Hartree‐Fock exchange (<0.2 eV). The SM‐corrected CLA approach also predicts 4.76 eV with a *D*_s_ correction of −1.06 eV, in reasonable agreement with QM/MM results. In Figure 3b, we plotted the intrinsic band alignment diagram among several MO_2_ oxides with respect to the vacuum level, where the bulk IP is calculated consistently at the BB1K level of theory using the QM/MM approach. This result shows the intrinsic band alignment with a complete exclusion of surface effects.

### Surface‐dependence of ionisation potentials

The periodic slab model (Figure 3c) considers both the bulk and surface contributions to the IP that can match the experimentally observed termination dependence.^[31]^ We calculated the IPs of CeO_2_ under different orientations using DFT at the PBE0 level of theory. A comparison with experimental measurements from Wardenga and Klein^[5]^ is given in Table S18. Unlike rock‐salt structured oxides that are dominated by the nonpolar (100) surface, the quadrupolar (111) surface (Figure 2c) is the most stable (*γ*=0.85 J m^−2^) in ceria with a high IP of 7.67 eV (Figure 2c). Wardenga and Klein reported an experimental IP of ca. 7.55 eV for the sample annealed at 700 °C in an O‐rich atmosphere, accompanied by 7.7 eV on the stoichiometric sample from Pfau and Schierbaum,^[7a]^ in excellent agreement with our results.

For the nonpolar CeO_2_(110) surface (Figure 2a), experiments on annealed samples observed that half of the surface atoms could be released while retaining surface stoichiometry, forming a terrace‐like reconstructed pattern (Figure 2b) composed of zigzagged (111) nanofacets.^[32]^ We confirmed by DFT calculations that this surface has an energy of 0.17 J m^−2^ lower than the unreconstructed (110) surface, suggesting an energetically favourable transformation at elevated temperatures. Upon reconstruction, the IP increases from 6.11 eV to 6.91 eV, a value lying between those of the standard (110) and (111) surfaces. Wardenga and Klein^[5]^ reported IPs of (110)‐oriented samples from 7.2 to 7.5 eV in O‐rich conditions, which could result from the energetically more favourable (111) nanofacets.

The polar CeO_2_(100) surface is intrinsically unstable unless appropriately reconstructed. An O‐terminated reconstructed pattern (Figure 2d) was first reported to be stabilised by removing half surface oxygen atoms for polarity compensation,^[33]^ with a calculated surface energy of 1.61 J m^−2^. Ce‐terminated (Figure 2f) CeO_2_(100) can also be synthesised on nanocube samples,^[34]^ although it is energetically less stable (*γ*=1.93 J m^−2^). Recently, López et al.^[35]^ discovered a pyramid‐like CeO_4_‐terminated configuration (Figure 2e) with a slightly lower surface energy (1.57 J m^−2^) than the O‐t pattern. The O‐t (100) surface has the highest calculated IP of 8.20 eV, while IPs of the CeO_4_‐t and Ce‐t reconstructed patterns are much lower (5.94 eV and 4.21 eV, respectively). The experimental IP reported by Wardenga and Klein ranges from 7.6 eV to 8.1 eV prepared in O‐rich conditions, where Ce‐terminated surfaces are less favourable.

Our current models do not consider the variable stoichiometry of ceria, which can further lead to more significant IP variations beyond the intrinsic surface effects. For example, fully reduced Ce_2_O_3_ could be considered as the upper limit of non‐stoichiometry effects in CeO_2−x_. Ce_2_O_3_ has two distinct lattice structures: a hexagonal phase (A‐type) and a cubic phase (C‐type).^[37]^ As will be discussed in detail in a future study, the reduction of CeO_2_ decreases the bulk $V_{Mad}^{O}$ from 21.73 V to 20.7 V in C‐Ce_2_O_3_ (with a calculated lattice constant of 11.200 Å at the PBE0 level of theory) and 20.10 V in A‐Ce_2_O_3_ (*a*=3.864 Å and *c*=6.088 Å from PBE0 calculations), indicating a lower bulk contribution to the IPs in reduced phases. Furthermore, the C‐Ce_2_O_3_(111) and A‐Ce_2_O_3_(001) surfaces, which maintain the CeO_2_(111) stacking sequence, have IPs of 6.53 eV and 7.19 eV (from O 2*p* states) calculated at the PBE0 level of theory, respectively. Compared with the 7.67 eV IP of CeO_2_(111), it can be estimated that variable stoichiometry in CeO_2−x_ could lead to a decrease in IP of up to 1.2 eV. The decrease in IP of CeO_2−x_ with an increasing percentage of Ce^3+^ is also observed experimentally by Wardenga and Klein.^[5]^

We further expand our analysis to other MO_2_‐type high‐*κ* dielectrics, including not only the cubic (*c*‐) phase but also the monoclinic (*m*‐) and tetragonal (t‐) phases of ZrO_2_ and HfO_2_. Like CeO_2_, the bulk contributes only 5.5–6.1 eV to their IPs (Figure 3b). Figure 3d shows the calculated IPs as a function of surface energies (Table S7–S9), with four regions highlighted to illustrate the similarity among these oxides. First, the most stable surfaces are all quadrupolar and exhibit relatively high IPs, ranging from the lowest 7.67 eV of CeO_2_(111) to the highest 8.32 eV of c‐HfO_2_(111). These results are consistent with experimental measurements using spectroscopic techniques (Table S1–S3), for example, 7.5–7.7 eV for CeO_2_(111),[^5^, ^7a^] 8.3–8.6 eV for ZrO_2_,^[36]^ and 8.0–8.4 eV for HfO_2_,^[37]^ where the most stable surfaces should be dominant. Next, nonpolar surfaces have much lower IPs, closer to the bulk values due to the lowest net surface dipoles. Then, reconstructions of polar surfaces result in considerable IP differences between the oxygen‐ and metal‐terminated patterns, which cover the highest (8.93 eV for O‐t c‐HfO_2_(100)) and lowest (4.21 eV for CeO_2_(100)Ce‐t) values. The remaining surfaces are mainly quadrupolar with a wide range of IPs and *γ* due to diverse stacking sequences.

We also calculated IPs for pristine surfaces of rutile (7.09–9.08 eV), anatase (7.20–8.88 eV), and brookite TiO_2_ (8.45–9.78 eV), as shown in Table S10. The small cation size in TiO_2_ increases the bulk $V_{Mad}^{O}$ to ca. 26 V, so that the bulk contribution to their IPs is much higher than CeO_2_, HfO_2_, and ZrO_2_ (Figure 3b). The lower cation polarisability (1.475 a.u.) further eliminates the IP discrepancies under different surface terminations.

Our calculations reveal that the IPs of MO_2_‐type high‐*κ* oxides are highly sensitive to their surface orientations. Differences in atomic stacking patterns, surface polarisation, and structural relaxation can result in variations of several electron volts in the IP, even for stoichiometric surfaces, which when combined with their variable surface chemistry, account for the uncertainties and controversies in energetic band alignment of metal oxides.^[25a]^ However, this diversity provides an opportunity to manipulate the band structure to meet specific requirements in technological applications. For instance, considering the close stability of O‐t and CeO_4_‐t CeO_2_(100)^[35]^ and over 2 eV difference in their IPs, their ratio during synthesis might be controlled to optimise the performance in band‐edge related chemical processes such as photocatalytic water‐splitting and hydration reactions.[^25b^, ^38^]

### The key role of electrostatics

**Figure 4:**
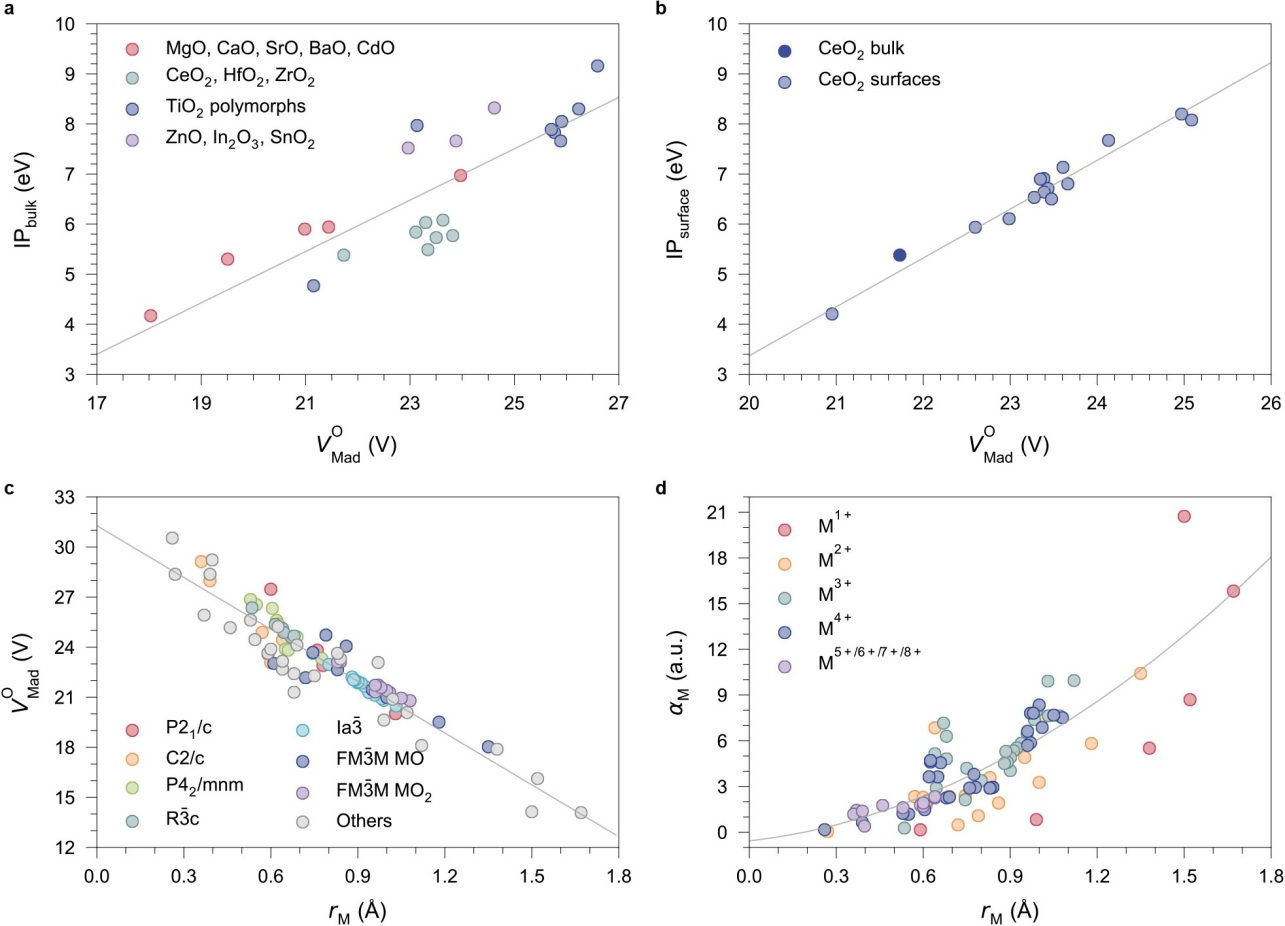
Impacts of the electrostatic environment and cation properties on the IPs of oxide materials. (a) Bulk IPs of various oxide materials as a function of the lowest in‐crystal Madelung potential on O sites ($V_{Mad}^{O}$ ). The bulk IPs of the rock‐salt structured oxides (MgO, CaO, SrO, BaO, and CdO) were previously determined by the SM‐corrected CLA method.^[19]^ Other data are calculated by the QM/MM approach at the BB1K level of theory, including transparent conducting oxides (ZnO, In_2_O_3_, and SnO_2_),^[30]^ TiO_2_ polymorphs,^[25]^ and CeO_2_, HfO_2_, and ZrO_2_ from this work. (b) Surface IPs (calculated by periodic slab models) of CeO_2_ as a function of $V_{Mad}^{O}$ . (c) The relationship between $V_{Mad}^{O}$ and Shannon effective radii^[12]^ of cations ($r_{M}$ ) in various binary oxides. (d) The relationship between cation polarisability ($\alpha_{M}$ ) and $r_{M}$ . Cations with the same formal charge states are shown using the same colour, and those with rare high oxidation states (M^5+^, M^6+^, M^7+^, and M^8+^) are shown together.

Figure 4a shows the relationship between the bulk IPs of various metal oxides and $V_{Mad}^{O}$ , reconfirming the critical role of electrostatics in determining the valence band edges. When multiple bonding environments exist for oxygen, the one with the lowest $V_{Mad}^{O}$ becomes the dominant site for ionisation. For example, baddeleyite TiO_2_ has a mix of twofold‐ and fourfold‐coordinated O ions with significantly different $V_{Mad}^{O}$ (21.15 V and 29.78 V, respectively). The calculated bulk IP is much lower (4.77 eV) than that of other TiO_2_ phases, and the anions at twofold‐coordinated sites are the preferential site for ionisation.^[25b]^ Despite considerable differences in oxidation states and crystal structures among these oxides, the classical treatment using Madelung potential shows the ability to predict variations in the bulk IP with reasonable accuracy. Surface IPs also correlate linearly with $V_{Mad}^{O}$ within a specific material. Slab models of CeO_2_ surfaces and their reconstructed configurations were employed to calculate the IPs and $V_{Mad}^{O}$ at surface O sites based on the SM. As shown in Figure 4b, the linear regression results in ${IP}_{surface}=0.976eV_{Mad}^{O}-16.139\;\;eV$ , with a coefficient of determination *R* ^2^ of 0.964, indicating that electrostatic interaction also determines surface band edge positions. As shown in Table S12, on most surfaces, the surface Ce atoms move further inwards into the bulk compared to oxygen, resulting in increased $V_{Mad}^{O}$ , and thus all stable O‐terminated surfaces of CeO_2_ contribute to a higher IP than the bulk. Additionally, except for (100)Ce‐t, the difference between the surface and bulk $V_{Mad}^{O}$ is negative, indicating an upward valence band bending near the surfaces.

The Madelung potential in metal oxides can be easily calculated using lattice energy codes such as GULP^[14]^ based on the given atomic structure and charge states. The linear relationship could also be used to assess the IPs of unknown systems with complex atomic structures. To further explore the interplay between the IPs of oxides with the properties of their constitutional ions, we calculated the bulk $V_{Mad}^{O}$ in 91 common binary oxides from Li_2_O to PuO_2_ within the point‐charge approximation, combined with gas‐phase cation polarisabilities calculated at the PBE0 level of theory. The full dataset is given in Table S19. Figure 4c shows the relationship of $V_{Mad}^{O}$ with Shannon ionic radii.^[12]^ We observed that, as expected, $V_{Mad}^{O}$ correlates in a reverse linear relationship with cation size. Therefore, oxides constituted with larger cations usually have lower $V_{Mad}^{O}$ and are expected to have lower bulk contributions to their IPs. However, as the size increases, the cation polarisability also becomes higher (Figure 4d). As a result, metal oxides with large cations are also expected to have higher surface contributions to their IPs because of the enhanced surface polarisation from cations. These conclusions could be extended to MO_2_‐ and M_2_O_3_‐type lanthanide and actinide oxides, where their IP is unknown. However, these oxides have similarly low bulk $V_{Mad}^{O}$ (ranging from 18.11 V of Ac_2_O_3_ to 22.21 V of Lu_2_O_3_) and relatively high cation polarisabilities (ranging from 4.17 a.u. of Lu^3+^ to 9.95 a.u. of Ac^3+^) as CeO_2_, thus should also show significant variations in the IPs under different surface terminations.

## Conclusion

Our study has deconvoluted the bulk and surface contributions to IPs of metal oxides using a range of theoretical techniques, emphasising the pivotal role of the electrostatic environment in determining the band edge positions. Our results reveal that apart from the well‐known near‐surface band bending, long‐range surface polarisation can significantly affect the absolute energy levels deep into the bulk, explaining the fundamental origins of IP variations in experimental measurements. We determined the bulk contribution to the IPs of CeO_2_, HfO_2_, and ZrO_2_ to be only 5.38–6.08 eV, while changing surface orientations can result in significant IP variations of several electron volts. Rational exploitation of these relationships could benefit the design of novel photovoltaic and electronic devices towards higher efficiencies.

## Conflict of interest

The authors declare no conflict of interest.

1

## Supporting information

As a service to our authors and readers, this journal provides supporting information supplied by the authors. Such materials are peer reviewed and may be re‐organized for online delivery, but are not copy‐edited or typeset. Technical support issues arising from supporting information (other than missing files) should be addressed to the authors.

Supporting Information

## Data Availability

The data that support the findings of this study are available from the corresponding author upon reasonable request.
