# Supplementary material for: Bulk and Surface Contributions to Ionisation Potentials of Metal Oxides
Source: Angew Chem Int Ed Engl. 2023 Aug 24;62(40):e202308411. doi: 10.1002/anie.202308411 (PMC10953407; doi:10.1002/anie.202308411)
Supplement: Supplementary file 1 — Supporting Information [file ANIE-62-0-s001.pdf]

Angewandte Chemie
Eine Zeitschrift der Gesellschaft Deutscher Chemiker

Supporting Information

Bulk and Surface Contributions to Ionisation Potentials of Metal Oxides

X. Zhang, T. Liu, L. Zhu, J. Guan, Y. Lu, T. W. Keal, J. Buckeridge, C. R. A. Catlow*, A. A. Sokol**

Table of Contents

1 Methods 3

1.1 Electrostatic analysis using shell-model potentials 3

1.2 Plane-wave DFT calculations 4

1.3 Hybrid QM/MM embedded-cluster approach 6

1.4 Polarisability calculation 8

2 Supporting Tables 9

3 Supporting Figure 24

4 References 25

1 Methods

1.1 Electrostatic analysis using shell-model potentials

Early in 1940, Mott and Gurney[1] proposed a semiempirical approach to evaluating the bulk IP of heteropolar solids based on the on-site electrostatic (Madelung[2]) potential (V_{Mad}). The calculation involves a periodic summation of the charges of surrounding ions for each crystallographic site within the point-charge approximation, *i.e.*, with the knowledge of only atomic coordinates and formal charges. V_{Mad} incorporates effects of the bulk chemical environment under 3D periodicity, avoids the uncertainty in how the electron escapes upon ionisation and allows for a direct comparison of on-site electrostatics in different systems. Based on this concept, we have taken the data on the lowest Madelung potentials on oxygen sites ($V_{\mathrm{Mad}}^{\mathrm{O}}$) in oxides reported by Walsh and Butler[3], calculated within the point-charge approximation, and supplemented with new data on lanthanide and actinide oxides, constituting a dataset covering 91 common binary oxides from Li_2O to PuO_2. In these calculations, we used experimentally measured lattice constants and atomic coordinates with references collected in Table S19.

With the aid of shell-model potentials, we further extend this approach to model oxide surfaces, showing that $V_{\mathrm{Mad}}^{\mathrm{O}}$ is also a reliable indicator for describing surface contributions to the IP. Compared with the point-charge approximation, the polarisable shell model[28] is an improved approximation for modelling heteropolar solids. A shell-model-based lattice-energy calculation describes the electrostatic Coulomb interaction using the Ewald summation technique,[4] while short-range repulsion and dispersion contributions are calculated using fitted interatomic potentials.[5] Based on a well-developed SM potential, one can simulate the formation of charged defects at the dilute limit using the Mott-Littleton approach,[6] and model surface properties using slab models under either two-dimensional (2D) or 3D periodicity,[7] as implemented in the GULP[8] code, for example.

In this work, we employed our previously developed SM potentials for CeO_2[6d] and rock-salt structured oxides[5] that reproduce the low-temperature structures and a wide range of physical properties. We cleaved ceria surfaces using the optimised bulk model with a lattice constant of 5.395 Å, which agrees well with the low-temperature measurement (5.401 Å at 100 K) and 0 K estimate extrapolated from thermal expansion measurements (*ca.* 5.395 Å).[6d, 9] In our original work, the connection between the core and shell is described by a harmonic spring:

$$E_{\mathrm{SM}}^{\mathrm{harmonic}} = \frac{1}{2} k_2 (\delta r_i)^2 \quad (1)$$

where k_2 is the harmonic spring constant, and δr_i is the core-shell distance. The spring constant and shell charges were fitted to reproduce the in-lattice ionic polarisability, which shows excellent agreement with QM/MM results in the calculated formation energies of charged

defects. However, atoms at the surface have lower coordination numbers compared to bulk. Shells at such undercoordinated surface sites could become over-polarised if the same spring constant is used, resulting in errors in the predicted surface structures. To improve the description of surface relaxation, in this work, we use a cosh spring function for the topmost surface atoms to replace the harmonic spring, which has the form of:

$$E_{\mathrm{SM}}^{\cosh} = k_2 d^2 \left(\cosh\left(\frac{\delta r_i}{d}\right) - 1\right) \tag{2}$$

where k_2 has the same value as the harmonic spring constant in equation (1) used for the bulk atoms, and parameter d is fitted separately for each surface due to their different atomic configurations. The optimised parameters are shown in Table S12, along with the predicted surface energies and atomic displacements on relaxation, compared with periodic DFT calculations. One-sided 2D-periodic surface models[10] are employed in SM-based calculations as implemented in GULP, in which the direction normal to the surface is non-periodic. The simulation cell is divided into two regions, where the positions of cores and shells in Region I can be optimised explicitly to model the surface relaxation, while those in Region II are fixed to reproduce the bulk environment. The surface energy γ is calculated as:

$$\gamma = \frac{E_{\mathrm{slab}}^{\mathrm{unrelax}} - E_{\mathrm{bulk}}}{2S} + \frac{E_{\mathrm{slab}}^{\mathrm{relax}} - E_{\mathrm{slab}}^{\mathrm{unrelax}}}{S} \tag{3}$$

where $E_{\mathrm{slab}}^{\mathrm{unrelax}}$ and $E_{\mathrm{slab}}^{\mathrm{relax}}$ are the total energies of the unrelaxed and relaxed surface models, respectively, E_{bulk} is the energy of the bulk containing the same number of atoms, and S is the surface area. For 2D surface slabs, the Coulomb sum is performed using the method proposed by Parry[11] to calculate the electrostatic potential at atomic sites. In each surface model, we placed a charge-neutral non-interacting probe "atom" at 40 Å above the surface to obtain the converged electrostatic potential in the vacuum layer. We employed relatively thick slab models (> 50 Å) in SM-based simulations to obtain fully converged surface energies (0.001 J m^{-2}) and electrostatic potentials (0.01 V). We observed no difference in the $V_{\mathrm{Mad}}^{\mathrm{O}}$ from bulk atoms based on a much thicker slab model (100 Å), suggesting a complete convergence in our calculations. With these settings, the predicted equilibrium surface structures and relative stabilities of CeO_2 surfaces described by our SM potential are generally in good agreement with DFT calculations (Table S12), although SM tends to overestimate the surface energies and atomic displacements on relaxation for some less-stable terminations.

1.2 Plane-wave DFT calculations

We conducted plane-wave DFT calculations using the Vienna Ab-initio Simulation Package (VASP)[12]. In 3D periodic models, the vacuum level is not clearly defined.[13] Instead, a stack of 2D slabs of finite thickness with a vacuum layer between the periodicity in the third dimension

is commonly employed to obtain the electrostatic Hartree potential in the near-surface vacuum region from the electron charge density. The surface IP is calculated by:

$$\mathrm{IP_s} = -eV_{\mathrm{vac}}^{\mathrm{s}} - \epsilon_{\mathrm{VBM}}^{\mathrm{s}} \tag{4}$$

where $V_{\mathrm{vac}}^{\mathrm{s}}$ is the average electrostatic potential at the central plane of the vacuum region, and $\epsilon_{\mathrm{VBM}}^{\mathrm{s}}$ is the energy level at the VBM.

As discussed above, surfaces can affect the positions of electronic band edges inside the bulk apart from near-surface band bending. Hence, while slab models have access to the vacuum, the energy levels in the centre of the slab are shifted by the long-range surface polarisation. To exclude surface effects and obtain the pure bulk contribution to the IP, a widely used remedy is the "core-level alignment" approach, which employs a core level, e.g., O 1*s* in oxides considered in this work, as a reference state to align the bulk energy levels calculated in a 3D periodic unit cell with the vacuum level obtained in a nonpolar surface model with fixed bulk geometry.[14] The bulk IP is therefore calculated as:

$$\mathrm{IP_b^{CLA}} = -eV_{\mathrm{vac}}^{\mathrm{s}} - \epsilon_{\mathrm{VBM}}^{\mathrm{b}} + \epsilon_{\mathrm{c}}^{\mathrm{b}} - \epsilon_{\mathrm{c}}^{\mathrm{s}} \tag{5}$$

where $V_{\mathrm{vac}}^{\mathrm{s}}$ is the electrostatic potential at the centre of the vacuum region, $\epsilon_{\mathrm{VBM}}^{\mathrm{b}}$ and $\epsilon_{\mathrm{c}}^{\mathrm{b}}$ are the VBM and O 1*s* core levels obtained in the bulk unit cell, and $\epsilon_{\mathrm{c}}^{\mathrm{s}}$ is the O 1*s* core level in the middle of the surface slab, assuming that this level is unperturbed by the surface.

This approach does not, however, fully consider surface-induced electronic polarisation, which plays a vital role in dielectric materials, as can be seen in the shift of $V_{\mathrm{Mad}}^{\mathrm{O}}$ in Fig. 1d-f from the "Fix" state to the "Shell" state. To address this issue, we have earlier proposed a strategy to assess the polarisation effect using SM potentials, which shows significant improvements in the estimated bulk IPs of metal oxides.[15] By comparing the electrostatic potential before and after shell relaxation using a nonpolar surface model with fixed bulk geometry, we are able to evaluate the shift of the VBM level when a surface is formed based on the multipolar shift D_{s}. This term is further used as a correction to DFT calculations, as given by:

$$D_{\mathrm{s}} = e\left(V_{\mathrm{vac}}^{\mathrm{shell}} - V_{\mathrm{vac}}^{\mathrm{fix}}\right) - e(V_{\mathrm{c}}^{\mathrm{shell}} - V_{\mathrm{c}}^{\mathrm{fix}}) \tag{6}$$

where $V_{\mathrm{vac}}^{\mathrm{shell}}$ and $V_{\mathrm{c}}^{\mathrm{shell}}$ are the electrostatic potentials on the non-interacting probe atom in the vacuum layer and on the O atom in the middle of the slab after the shell relaxation, respectively, and $V_{\mathrm{vac}}^{\mathrm{fix}}$ and $V_{\mathrm{c}}^{\mathrm{fix}}$ are the corresponding values before shell relaxation at the "Fix" state. Therefore, the IP calculated by the SM-corrected CLA approach is:

$$\mathrm{IP_b^{SM-CLA}} = \mathrm{IP_b^{CLA}} + D_s \tag{7}$$

By adding the D_s correction to $\mathrm{IP_b^{CLA}}$ from DFT calculations, the vacuum level and VBM are shifted back to unpolarised positions before the electronic redistribution, which excludes surface effects in calculating the bulk IP.

In our spin-polarised VASP calculations, a plane-wave basis set with a cut-off energy of 600 eV has been employed to describe the valence electronic states along with the projector augmented wave (PAW)[16] method to describe interactions between the core and valence electrons. The valence states considered in this study included Ce (5*s*, 5*p*, 4*f*, 5*d*, 6*s*), Hf (6*s*, 5*d*), Zr (4*s*, 4*p*, 5*s*, 4*d*), Ti (3*d*, 4*s*), Mg (2*s*), Ba (5*s*, 5*p*, 6*s*), and O (2*s*, 2*p*) states. Monkhorst-Pack *k*-point meshes were generated with a density of 0.04 × 2π $Å^{-1}$ for bulk and surface systems. The hybrid exchange and correlation density functional PBE0[17] was employed with a 25% non-local Hartree-Fock exchange for modelling pristine surfaces, which shows excellent performance in modelling defects and surfaces, as shown in our previous work on CeO_2.[6d] A detailed comparison of the predicted lattice parameters, band gaps, and dielectric constants of the studied ZrO_2, HfO_2, and TiO_2 systems with experimental measurements is given in Table S4-S6. The optimised unit cells at the PBE0 level of theory were further used to construct the QM/MM models for ZrO_2 and HfO_2. The theoretical band gap reported in this work in determining the intrinsic band alignment of MO_2 oxides was calculated using the HSE06 hybrid functional,[18] which shows better agreement with experiment for semiconductors and insulators.[19] The optimised lattice parameters using the PBE0 and HSE06 functionals are almost identical in all studied systems with less than 0.02% differences.

The (100), (110), and (111) surface models of CeO_2 were adopted from our previous work,[6d] in which the central atomic layer was fixed, while other atoms on the two sides were allowed to relax. Such a three-region model results in consistent surface energies with the two-region model. A 30-Å-thickness vacuum layer was used in all DFT slab models, ensuring full convergence of surface energies and electrostatic potentials. Similar settings were used to model other pristine surfaces, and a comparison of the calculated surface energies with other theoretical studies is presented in Table S7-S10.

1.3 Hybrid QM/MM embedded-cluster approach

The central problem in theoretical calculations of band alignment of different materials is to define a common reference. The hybrid QM/MM embedded-cluster approach has been developed as a state-of-the-art theoretical method to calculate the bulk IP of ionic solids that excludes any surface effect.[20] In a solid-state QM/MM model, a part of the crystal is treated with a QM method, such as hybrid DFT, embedded in the remaining lattice described by interatomic potential techniques. With the description of SM potentials, the surrounding regions can reproduce effectively the bulk long-range electrostatic environment and account for accurate electronic polarisation, including the dielectric response to the ionisation process.[20d] Furthermore, QM/MM models allow direct access to the vacuum level by excluding all electrostatic contributions at surfaces, using a frozen-MM region and fitted point charges outside the active regions to reproduce the bulk electrostatic environment. This approach has

been implemented in the ChemShell package[20a, 20b, 21] and widely employed to predict the bulk IPs of many oxides, nitride, and sulfide systems.[6b, 20c, 22]

In the QM/MM approach, the bulk IP is calculated by the energy difference between an electronically relaxed ionised system ($q = +1$) and a charge-neutral system:

$$\mathrm{IP}_{\mathrm{b}}^{\mathrm{QM/MM}} = (E_{q=+1} + E_{\mathrm{corr}}) - E_{q=0} \tag{8}$$

The long-range polarisation effect in charged systems is treated with an *a posteriori* correction on the calculated QM/MM energy, as proposed by Jost[20b, 23]:

$$E_{\mathrm{corr}} = -\frac{Q^2}{2R}(1 - \frac{1}{\varepsilon_\infty}) \tag{9}$$

where R is the radius of the active region (15 Å in this work), Q is the net charge of the system, and ε_∞ is the high-frequency dielectric constant of the material.

In this work, QM/MM calculations of the bulk IPs were performed with the python-based version of ChemShell (Py-ChemShell),[21] with NWChem[24] and GULP[8] as the QM and MM drivers, respectively. We employed the QM/MM model of CeO_2 developed in our previous work.[6d] The interatomic potentials for HfO_2 and ZrO_2 polymorphs are designed to only reproduce the high-frequency dielectric constants at the fixed PBE0 geometry (*i.e.*, only shell-relaxation is permitted, while core positions remain fixed at the PBE0 geometry). The predicted static dielectric constants are however still reasonable, as shown in Table S4-S5 in the SI, compared with experimental measurements. The full SM parameters are given in Table S13 and S14. The QM/MM models for ZrO_2 and HfO_2 polymorphs were constructed using newly fitted SM potentials based on the fixed PBE0 geometry, while only electronic degrees of freedom are allowed to relax, following the same procedures as our previous models for TiO_2 polymorphs.[20c, 22c] The Def2-TZVP basis set[25] and the effective core potentials (ECPs) from Andrae *et al.*[26] were used in the QM calculations. Hybrid functionals mitigate some of the self-interaction error inherent in semilocal functionals, which are expected to improve the accuracy of energy level predictions in metal oxides. In our QM/MM calculations, we mainly employed the hybrid meta-generalized-gradient approximation (meta-GGA) functional BB1K with 42% exact exchange,[27] which provides an accurate description of thermochemical kinetics, as well as the localisation of electron and hole polarons in metal oxides. Results obtained from hybrid GGA functionals B97-2[28], PBE0[17] and HSE06[18] are also shown for comparison in Table S15. PBE0 with a 25% of exact exchange is a widely applicable hybrid functional for metal oxides. HSE06 is developed based on the same formula while further incorporating range-separation parameters to consider the screening effect. B97-2, similar to BB1K, was derived purely based on thermochemical data but has a significantly lower percentage of exact exchange (21%).

For the QM/MM interfaces, specially designed local pseudopotentials are placed on cations, fitted to minimise the QM/MM mismatch and align the deep core levels at symmetry-equivalent positions. Detailed settings in the QM/MM calculations are given in Table S11 of the SI.

1.4 Polarisability calculation

Calculations of gas-phase and in-lattice ionic polarisabilities were performed using NWChem at the PBE0 level of theory with the Def2-QZVP basis set,[25, 29] following the same procedures presented in our previous work.[6d]

2 Supporting Tables

Table S1. Summary of experimental measurements and theoretical calculations of the ionisation potential (IP), electron affinity (EA), band gap (E_g), and work function (Φ) of CeO_2.

Sample	Methods	IP (eV)	EA (eV)	E_g (eV)	Φ (eV)
CeO_2(100) films[a]	XPS, UPS	7.1-9.1			4.1-6.3
CeO_2(110) films[a]		6.5-9.0			3.5-6.3
CeO_2(111) films[a]		6.5-8.6			3.5-5.8
CeO_2(111) films[b]	XPS, UPS, EELS	7.7	3.3	4.4	4.7
CeO_2/$CeO_{2-\delta}$ core-shell particles[c]	XPS, UPS, EELS	5.47 (CeO_2)	1.82	3.65	
		5.74 ($CeO_{2-\delta}$)	2.32	3.42	
Octahedral particles[d]	Mott-Schottky	7.69 (52 nm)	4.27	3.42	
	measurement, UV-	7.56 (85 nm)	4.45	3.11	
	vis	7.39 (110 nm)	4.45	2.94	
CeO_{2-x}-based heterojunctions[e]	XPS, KPFM, UV-vis	7.21	4.46	2.75	4.69
Al/CeO_2/Si[f]	Band alignment	6.86	3.63	3.23	
Al/CeO_2[g]	Band alignment	6.94	3.62	3.32	
Al/CeO_2/n-Si[h]	Band alignment		3.40		
Al/CeO_2[i]	Band alignment	6.82	3.49	3.33	
Ag decorated CeO_2 nanorod[j]	Band alignment	7.16	3.5	3.66	
CeO_2(111) film on Si[k]	XPS	7.4			
Au/amorphous CeO_2/Au[l]	Band alignment	7.17	4.17	3.0	
Polycrystalline CeO_2/Ge[m]	Band alignment	6.59	2.99	3.6	
CeO_2 nanocrystals/SiO_2[n]	Band alignment	6.75	3.6	3.15	
Au/Ni/CeO_2/GaN[o]	Band alignment	6.99	3.47	3.52	
CeO_2/Ag_2O[p]	Mott-Schottky measurement	6.82	4.1	2.72	
CeO_2(111)[q]	PBEsol+U	6.44	4.27	2.17	5.45
CeO_2(111)[r]	DFT-D3, PBE	6.48	3.0	6.18	6.48
CeO_2(111)[s]	HSE (α = 0.18)	6.87	3.56	3.31	
CeO_2[t]	Analytical model	3.93	0.78	3.15	

[a] CeO_2 thin films prepared by radio frequency magnetron sputter deposition under different conditions.[30]

[b] Stoichiometric CeO_2(111) thin film samples.[31]

[c] Ref. [32]

[d] Ref. [33]

[e] Ref. [34]

[f-i] Measurement of Schottky barriers of the Al/CeO_2 interfaces (0.62 eV,[35] 0.63 eV,[36] 0.85 eV,[37] and 0.76 eV[38]), and Φ_{Al} = 4.25 eV[39].

[j] Band alignment with Ni with a Schottky barrier of 0.76 eV,[40] and Φ_{Ag} = 4.26 eV[39].

[k] Band alignment with Si with a valence band offset (VBO) of 2.2 eV,[41] and IP_{Si} = 5.2 eV[42].

[l] Band alignment with Au with a Schottky barrier of 0.93 eV,[40] and Φ_{Au} = 5.1 eV[39].

m Band alignment with Ge with a VBO of 1.5 eV,[43] and IP_{Ge} = 5.09 eV[44].

n Band alignment with SiO_2 with a VBO of -2.95 eV,[41] and IP_{SiO2} = 9.7 eV[42].

o Band alignment with Ni with a Schottky barrier of 1.68 eV,[40] and Φ_{Ni} = 5.15 eV[39].

p The VBM of CeO_2 is measured at 2.38 eV below the normal hydrogen electrode (NHE, -4.44 eV).[45]

q Ref. [46]

r Ref. [47]

s Ref. [48]

t Analytic band alignment with Si with a conduction band offset (CBO) of 2.7 eV,[49] and EA_{Si} = 4.08 eV[42]

Table S2. Summary of experimental measurements and theoretical calculations of the ionisation potential (IP), electron affinity (EA), band gap (E_g), and work function (Φ) of HfO_2.

Sample	Methods	IP (eV)	EA (eV)	E_g (eV)	Φ (eV)
Ge(100)/HfO_2[a]	Band alignment	8.09	2.49	5.6	
HfO_2/SiO_2/Si[b]	Resonant photoelectron spectroscopy	8.4	2.2	6.2	5.0
HfO_2/Si(Ge)[c]	XPS, UPS	7.97	2.17	5.8	
a-HfO_2/Si[d]	Band alignment	7.94 (a-HfO_2)	2.44	5.5	
m-HfO_2/Si[d]	Band alignment	7.65 (m-HfO_2)	1.75	5.9	
HfO_2/Si(100)[e]	Band alignment	8.06	2.56	5.5	
HfO_2/SiO_2/Si[f]	Band alignment	8.4 (m-HfO_2)	2.76	5.64	
a-HfO_2/Si[g]	Band alignment	7.7	2.2	5.5	
Si/HfO_2[h]	DFT (GGA)	9.34 (O-rich)<br>9.16 (Hf-rich)			
Charge-neutrality-level model[i]	Tight-binding	8.6			
MO_2 heterojuctions[j]	DFT (HSE/-U)	8.33			
m-HfO_2 (Interstitial H level)[k]	DFT (HSE)	8.7		5.86	

a Band alignment with Ge with a VBO of 3.0 eV,[50] and IP_{Ge} = 5.09 eV[44].

b Ref. [51]

c Ref. [52]

d-g Band alignment with Si with a VBO of 2.74 eV,[53] 2.45 eV,[53] 2.86 eV,[54] 3.20 eV,[55] 2.5 eV,[56] and IP_{Si} = 5.2 eV[42].

h Ref. [57]

i Ref. [58]

j Band alignment with rutile TiO_2 with a VBO of 0.5 eV,[59] and $IP_{r\text{-}TiO2}$ = 7.83 eV[20c].

k Ref. [60]

Table S3. Summary of experimental measurements and theoretical calculations of the ionisation potential (IP), electron affinity (EA), band gap (E_g), and work function (Φ) of ZrO_2.

Sample	Methods	IP (eV)	EA (eV)	E_g (eV)	Φ (eV)
Yttria-stabilised zirconia (YSZ, 10% Y_2O_3)[a]	XPS, UPS, EELS	8.3	3.1	5.2	4.9

ZrO_2/C_{60}[b]	UPS	8.6	2.9	5.7	3.3-4.2
c-ZrO_2/Si[c]	Band alignment	7.53			
c-ZrO_2/Si[d]	Band alignment	7.55		5.3	
Al_2O_3/ZrO_2/Si[e]	Band alignment	7.7		5.6	
ZrO_2/Si[f]	Band alignment	8.07		5.18	
t-ZrO_2/Si[g]	Band alignment	8.34		5.3	
ZrO_2/Si[h]	Band alignment	8.35		5.5	
Si/ZrO_2[i]	DFT (GGA)	9.28 (O-rich)			
		9.11 (Zr-rich)			
Charge-neutrality-level model[j]	Tight-binding	8.5			
t-ZrO_2/Si(100)[k]	DFT (LDA)	9.09		3.9	
MO_2 heterojuctions[l]	DFT (HSE/-U)	8.43		5.54	
m-ZrO_2 (Interstitial H level)[m]	DFT (HSE)	8.7		5.22	

[a] Measured at 700 K in contact with Fe/FeO.[61]

[b] Ref. [62]

[c-h] Band alignment with Si with a VBO of 2.33 eV,[53] 2.35 eV,[54] 2.5 eV,[63] 2.87 eV,[64] 3.14 eV,[55] 3.15 eV,[65] and IP_{Si} = 5.2 eV[42].

[i] Ref. [57]

[k] Ref. [58]

[l] Band alignment with rutile TiO_2 with a VBO of 0.6 eV,[59] and $IP_{r\text{-}TiO2}$ = 7.83 eV[20c].

[m] Ref. [60]

Table S4. DFT-calculated lattice parameters (a_0), band gaps (E_g), and static (ε_0) and high-frequency (ε_∞) dielectric constants for ZrO_2 compared with experimental measurements. The calculated dielectric constants by fitted shell-model potentials based on fixed PBE0 geometry (only shell relaxation is allowed) are also shown.

	PBE0	HSE06	Shell model	Experiment
**(a) c-ZrO_2**				
a_0 (Å)	5.073	5.074		5.09 (0K)[66]
E_g (eV)	5.79	5.02		6.1[67]
ε_0			27.72	27.2,[68] 29.3[69]
ε_∞	4.86	4.89	4.78	4.61,[67] 4.67[70]
**(b) t-ZrO_2**				
a_0 (Å)	3.590	3.592		3.607[67]
c_0 (Å)	5.195	5.198		5.181[67]
E_g (eV)	6.482	5.74		5.78[67]
$\varepsilon_{0,a/c}$			$34.94_a/29.14_c$	34.5,[68] 39.8[69] (macroscopic results, same below)
$\varepsilon_{\infty,a/c}$	$4.87_a/4.42_c$	$4.89_a/4.43_c$	$4.67_a/4.51_c$	4.805-4.875[67]

**(c) m-ZrO_2**				
a_0 (Å)	5.144	5.138		5.145[71]
b_0 (Å)	5.209	5.217		5.207[71]
c_0 (Å)	5.309	5.307		5.311[71]
β (°)	99.46	99.59		99.23[71]
E_g (eV)	6.04	5.27		5.83[67]
$\varepsilon_{0,a/b/c}$			$18.30_a/25.21_b/27.12_c$	16,[72] 24[73]
$\varepsilon_{\infty,a/b/c}$	$4.57_a/4.58_b/4.33_c$	$4.59_a/4.60_b/4.35_c$	$4.48_a/4.61_b/4.44_c$	4.80[72]

Table S5. DFT-calculated lattice parameters (a_0), band gaps (E_g), and static (ε_0) and high-frequency (ε_∞) dielectric constants for HfO_2 compared with experimental measurements. Shell-model prediction of dielectric constants with fixed PBE0 geometry is also shown.

	**PBE0**	**HSE06**	**Shell model**	**Experiment.**
**(a) c-HfO_2**				
a_0 (Å)	5.023	5.023		5.08[74]
E_g (eV)	6.14	5.33		5.8[75]
ε_0			25.30	27[76]
ε_∞	4.40	4.43	4.61	4.46[77]
**(b) t-HfO_2**				
a_0 (Å)	3.554	3.555		3.603[78]
c_0 (Å)	5.130	5.131		5.121[78]
E_g (eV)	6.95	6.18		5.5,[79] 5.8[80]
$\varepsilon_{0,a/c}$			$29.89_a/25.21_c$	35,[79] 36[81]
$\varepsilon_{\infty,a/c}$	$4.41_a/4.09_c$	$4.44_a/4.11_c$	$4.57_c/4.35_a$	3.5,[82] 3.8[83]
**(c) m-HfO_2**				
a_0 (Å)	5.092	5.095		5.116[71]
b_0 (Å)	5.160	5.164		5.172[71]
c_0 (Å)	5.248	5.248		5.295[71]
β (°)	99.59	99.60		99.18[71]
E_g (eV)	6.39	5.58		5.68[84]
$\varepsilon_{0,a/b/c}$			$18.73_a/24.57_b/23.97_c$	16[85]
$\varepsilon_{\infty,a/b/c}$	$4.18_a/4.17_b/3.97_c$	$4.20_a/4.18_b/3.99_c$	$4.40_a/4.53_b/4.34_c$	4[58]

Table S6. DFT-calculated lattice parameters (a_0), band gaps (E_g), and static (ε_0) and high-frequency (ε_∞) dielectric constants for TiO_2 polymorphs compared with experimental measurements.

	**PBE0***	**HSE06***	**Expt.**
**(a) r-TiO_2**			

a_0 (Å)	4.599	4.560	4.593[86]
b_0 (Å)	4.599	4.560	4.593[86]
c_0 (Å)	2.947	2.947	2.959[86]
E_g (eV)	4.00	3.26	3.031[87]
ε_0			$111_a/257_c$,[88]
			$114.9_a/251_c$[89]
ε_∞	$6.07_a/7.31_c$	$6.12_a/7.37_c$	$6.84_a/8.83_c$,[90]
			6.33,[91]
			6.81[92]
**(b) a-TiO_2**			
a_0 (Å)	3.779	3.780	3.796[93]
b_0 (Å)	3.779	3.780	3.796[93]
c_0 (Å)	9.608	9.609	9.444[93]
E_g (eV)	4.11	3.39	3.23[94]
ε_0			$45.1_a/22.7_c$,[95]
			31,[96]
			45[97]
ε_∞	$5.75_a/5.48_c$	$5.80_a/5.50_c$	$5.82_a/5.41_c$,[95]
			5.62,[91]
			6.20[92]
**(c) b-TiO_2**			
a_0 (Å)	9.186	9.192	9.174[86]
b_0 (Å)	5.444	5.445	5.449[86]
c_0 (Å)	5.138	5.140	5.138[86]
E_g (eV)	4.26	3.51	3.1-3.4[98]
ε_0			78,[96]
			93[99]
ε_∞	$6.31_a/5.90_b/5.92_c$	$6.36_a/5.96_b/5.97_c$	5.91-6.55,[100]
			6.66[92]

* This work.

Table S7. Models of stoichiometric CeO_2 surfaces employed in periodic DFT calculations, their ionisation potentials (IP), work functions (Φ), and surface energies (γ) calculated at the PBE0 level of theory. Previous theoretical results of surface energies are also shown for comparison when available.

**Surface**	**Type**	**IP (eV)***	**Φ (eV)***	**γ (J m^{-2})***	**γ (J m^{-2})**	**γ (J m^{-2})**	**γ (J m^{-2})**
(100)O-t[101]	polar	8.20	8.19	1.61	1.64[a]	1.64[b]	1.709[c]
(100)Ce-t[101]	polar	4.21	3.89	1.93		1.83[b]	1.916[c]

(100)CeO_4-t[101]	polar	5.94	5.68	1.57			1.634[c]
(110)	nonpolar	6.11	5.84	1.21	1.27[a] [102]	1.09[b]	
(110)r[103]	nonpolar	6.91	6.59	1.04			
(111)	quadrupolar	7.67	7.67	0.85	0.86[a]	0.73[b]	
(111)r[104]	quadrupolar	8.08	7.83	2.01			
(210)	polar	6.80	6.58	1.31			
(211)	nonpolar	6.71	6.40	1.40			
(221)	polar	7.14	6.80	0.89		0.88[b]	
(311)	quadrupolar	6.50	6.25	1.63			
(321)	nonpolar	6.64	6.31	1.24			
(331)	quadrupolar	6.90	6.66	1.03		0.95[b]	
(531)	quadrupolar	6.53	6.26	1.35			

* This work

[a] Hybrid PBE0 results from Ref. [102]

[b] PBE+U ($U_{Ce\ 4f}$ = 5 eV) results from Ref. [105]

[c] Hybrid HSE06 results with zero point vibrational energy contributions from Ref. [101]

Table S8. Models of stoichiometric ZrO_2 surfaces employed in periodic DFT calculations, their ionisation potentials (IP), work functions (Φ), and surface energies (γ) calculated at the PBE0 level of theory. Previous theoretical results of surface energies are also shown for comparison when available.

**Surface**	**Type**	**IP (eV)***	**Φ (eV)***	**γ (J m^{-2})***	**γ (J m^{-2})**	**γ (J m^{-2})**
c-ZrO_2(100)O-t	polar	8.79	8.53	1.16		
c-ZrO_2(100)ZrO_4-t	polar	5.57	5.23	1.42		
c-ZrO_2(110)	nonpolar	6.23	5.85	1.42	1.04[a]	
c-ZrO_2(111)	quadrupolar	8.31	7.97	0.91	0.769[a]	
t-ZrO_2(001)	polar	8.78	8.42	1.29		1.17[c]
t-ZrO_2(100)	nonpolar	6.93	6.59	1.31		1.23[c]
t-ZrO_2(110)	polar	8.31	7.97	1.65		1.77[c]
t-ZrO_2(101)	quadrupolar	8.22	7.91	1.15		1.06[c]
t-ZrO_2(111)	quadrupolar	7.61	7.21	1.45		1.25[c]
m-ZrO_2(001)	quadrupolar	8.43	8.20	1.49	1.31[b]	1.35[d]
m-ZrO_2(010)	quadrupolar	8.82	8.48	1.88	1.65[b]	1.75[d]
m-ZrO_2(100)	quadrupolar	8.66	8.26	1.65	1.46[b]	1.47[d]
m-ZrO_2(110)	quadrupolar	7.14	6.76	1.38	1.22[b]	1.42[d]
m-ZrO_2(101)	quadrupolar	7.28	7.02	1.60	1.42[b]	1.52[d]
m-ZrO_2(011)	quadrupolar	7.33	6.93	1.49	1.34[b]	1.34[d]

m-ZrO_2($\bar{1}01$)	quadrupolar	7.84	7.58	1.38	1.23[b]	1.23[d]
m-ZrO_2(111)	quadrupolar	8.22	7.90	1.25	1.11[b]	1.06[d]
m-ZrO_2($\bar{1}11$)	quadrupolar	7.97	7.68	1.06	0.94[b]	0.87[d]

* This work

[a] PBE results from Ref. [106]

[b] PBE results from Ref. [107]

[c] PW91 results from Ref. [108]

[d] PW91 results from Ref. [109]

Table S9. Surface models of stoichiometric HfO_2 employed in periodic DFT calculations, their ionisation potentials (IP), work functions (Φ), and surface energies (γ) calculated at the PBE0 level of theory. Previous theoretical results of surface energies are also shown for comparison when available.

**Surface**	**Type**	**IP (eV)***	**Φ (eV)***	**γ (J m^{-2})***	**γ (J m^{-2})**	**γ (J m^{-2})**
c-HfO_2(100)O-t	polar	8.93	8.68	1.34		
c-HfO_2(100)HfO_4-t	polar	5.42	5.19	1.74		
c-HfO_2(110)	nonpolar	6.16	5.93	1.56		
c-HfO_2(111)	quadrupolar	8.32	8.09	1.00		
t-HfO_2(001)	polar	8.92	8.63	1.45		1.21[b]
t-HfO_2(100)	nonpolar	6.90	6.64	1.39		1.55[b]
t-HfO_2(110)	polar	8.70	8.40	1.79		1.08[b]
t-HfO_2(101)	quadrupolar	8.27	7.96	1.24		1.54[b]
t-HfO_2(111)	quadrupolar	7.74	7.51	1.53		1.12[b]
m-HfO_2(001)	quadrupolar	8.53	8.29	1.73	1.416[a]	1.51[b]
m-HfO_2(010)	quadrupolar	8.65	8.65	2.14	1.878[a]	1.88[b]
m-HfO_2(100)	quadrupolar	8.70	8.48	1.89	1.667[a]	1.67[b]
m-HfO_2(110)	quadrupolar	7.16	6.89	1.58	1.388[a]	1.38[b]
m-HfO_2(101)	quadrupolar	7.33	7.07	1.80	1.550[a]	1.57[b]
m-HfO_2(011)	quadrupolar	7.42	7.11	1.67	1.484[a]	
m-HfO_2($\bar{1}01$)	quadrupolar	7.92	7.69	1.54	1.322[a]	
m-HfO_2(111)	quadrupolar	8.31	8.00	1.42	1.199[a]	1.25[b]
m-HfO_2($\bar{1}11$)	quadrupolar	7.99	7.75	1.19	0.993[a]	

* This work

[a] PW91 results from Ref. [110]

[b] PBE results from Ref. [111]

Table S10. Models of stoichiometric TiO_2 surfaces employed in periodic DFT calculations, their ionisation potentials (IP), work functions (Φ), and surface energies (γ) calculated at the PBE0 level of theory. Previous theoretical results of surface energies are also shown for comparison when available.

Surface	Type	IP (eV)*	Φ (eV)*	γ (J m^{-2})*	γ (J m^{-2})	γ (J m^{-2})	γ (J m^{-2})	γ (J m^{-2})	γ (J m^{-2})
r-TiO_2(100)	quadrupolar	9.08	8.85	0.83	0.67[a]	0.94[b]	0.833[d]	0.85[f]	
r-TiO_2(001)	nonpolar	7.09	6.86	1.52	1.21[a]	1.59[b]	1.587[d]	1.47[f]	
r-TiO_2(110)	quadrupolar	8.85	8.56	0.66	0.48[a]	0.67[b]	0.595[d]	0.62[f]	
r-TiO_2(101)	quadrupolar	8.12	7.84	1.20	1.01[a]	1.19[b]		1.20[f]	
r-TiO_2(111)	quadrupolar	7.71	7.41	1.64					
a-TiO_2(100)	nonpolar	8.51	8.29	0.73	0.58[c]		0.732[d]		
a-TiO_2(001)	quadrupolar	7.67	7.38	1.24	0.98[c]			1.36[f]	
a-TiO_2(110)	nonpolar	8.25	7.95	1.26	1.15[c]			1.38[f]	
a-TiO_2(101)	quadrupolar	8.88	8.59	0.62	0.49[c]		0.621[d]	0.64[f]	
a-TiO_2(111)	quadrupolar	7.20	6.90	1.99					
b-TiO_2(001)	quadrupolar	9.78	9.49	0.92				1.47[f]	0.62[g]
b-TiO_2(010)	quadrupolar	8.67	8.37	0.99		0.97[e]		1.05[f]	0.77[g]
b-TiO_2(100)	quadrupolar	9.20	8.94	1.07		1.27[e]		1.17[f]	0.88[g]
b-TiO_2(110)	quadrupolar	8.65	8.35	0.99		1.16[e]		1.07[f]	0.85[g]
b-TiO_2(101)	quadrupolar	8.45	8.22	1.15					0.87[g]
b-TiO_2(011)	quadrupolar	8.69	8.45	0.98					0.85[g]
b-TiO_2(111)	quadrupolar	8.81	8.59	0.90					0.75[g]
b-TiO_2(210)	quadrupolar	8.69	8.42	0.82					0.70[g]

* This work

[a] PBE results from Ref. [112]

[b] Hybrid B3LYP results from Ref. [113]

[c] PBE results from Ref. [114]

[d] Hybrid PBE0 results from Ref. [115]

[e] Hybrid B3LYP results from Ref. [116]

[f] Hybrid PW1PW results from Ref. [117]

[g] GGA results from Ref. [118]

Table S11. Computational Details in the QM/MM calculations of bulk ionisation potentials of MO_2 oxides.

	CeO_2	c-ZrO_2, t-ZrO_2, m-ZrO_2	c-HfO_2, t-HfO_2, m-HfO_2
**(a) QM calculations**			

ECP for M^{4+}	Ref. [119] (28 core e^-)	Ref. [26] (28 core e^-)	Ref. [26] (60 core e^-)
Basis set for M^{4+}	Ref. [120]	Def2-TZVP[25]	Def2-TZVP[25]
Basis set for O^{2-}	Def2-TZVP[25]	Def2-TZVP[25]	Def2-TZVP[25]
No. of QM atoms	197	111_{c-}, 111_{t-}, 101_{m-}	111_{c-}, 111_{t-}, 101_{m-}
**(b) Embedding cation ECP parameters for the QM/MM interface**			
$$U_p(r) = \frac{1}{r^2}(A_1\, r\, e^{-Z_1\, r^2} + A_2\, r^2\, e^{-Z_2\, r^2} + A_3\, r^2\, e^{-Z_3\, r^2})$$			
A_1 (a.u.)	-49.817	-44.817	-44.817
Z_1 (a.u.)	24.9589	25.5589	25.6389
A_2 (a.u.)	60.247	59.803	59.603
Z_2 (a.u.)	2.85901	2.85901	2.85961
A_3 (a.u.)	0.295877	0.280667	0.288667
Z_3 (a.u.)	0.25855	0.323486	0.333276
**(c) Force fields in the MM regions**			
	Ref. [6d]	Table S13	Table S14

Table S12. Comparison of the optimised surface structures and surface energies (γ) of CeO_2 predicted by shell-model-based slab simulations, compared with DFT calculations at the PBE0 level of theory. For each surface, the cosh spring parameters (d_O and d_{Ce}) were fitted to reproduce the surface relaxation predicted by DFT, as described in the Method section in the main text. $\Delta_{O(z)}$ and $\Delta_{Ce(z)}$ indicate the displacements of the topmost surface atoms normal to the surface after structural relaxation.

	**Shell-model**					**DFT PBE0**		
**Surface**	d_O **(Å)**	d_{Ce} **(Å)**	γ **(J m^{-2})**	$\Delta_{O(z)}$ **(Å)**	$\Delta_{Ce(z)}$ **(Å)**	γ **(J m^{-2})**	$\Delta_{O(z)}$ **(Å)**	$\Delta_{Ce(z)}$ **(Å)**
(111)	0.032	0.001	0.96	-0.034	-0.017	0.85	-0.034	-0.012
(221)	0.5	0.03	1.02	-0.047	-0.152	0.89	-0.049	-0.156
(331)	0.5	0.03	1.15	-0.063	-0.190	1.03	-0.053	-0.175
(110)r[103]	0.5	0.015	1.17	-0.091	-0.270	1.04	-0.079	-0.228
(321)	0.5	0.01	1.64	0.006, -0.075	-0.315	1.24	0.006, -0.039	-0.174
(110)	0.8	0.01	1.73	-0.018	-0.201	1.21	-0.022	-0.163
(210)	0.3	0.08	1.79	-0.204	-0.193	1.31	-0.140	-0.160
(531)	0.5	0.015	1.82	-0.085	-0.291	1.35	-0.051	-0.178
(211)	0.5	0.03	1.88	-0.056, -0.149	-0.285	1.40	-0.001, -0.039	-0.195
(100)CeO_4-t[101]	0.5	0.06	2.22	-0.348, -0.015, 0.029	-0.410	1.57	-0.302, 0.024, 0.041	-0.403

(100)O-t[101]	0.3	0.0035	2.56	-0.279	0.101	1.61	-0.278	0.078
(311)	0.5	0.01	2.57	-0.150	-0.311	1.63	-0.088	-0.181
(100)Ce-t[101]	0.3	0.08	2.83	-0.034	-0.411	1.93	0.010	-0.404
(111)r[104]	0.5	0.03	3.03	-0.423	-0.168, 0.069	2.01	-0.505	-0.200, 0.011

Table S13. Shell-model interatomic potential derived for ZrO_2

(a) Buckingham potential

$$V_{ij}(r_{ij})^{\text{Buckingham}} = A\exp\left(-\frac{r_{ij}}{\rho}\right) - C_6 r_{ij}^{-6}$$

**Interaction**	***A*** **(eV)**	***ρ*** **(Å)**	***C*₆ (eV Å⁶)**	***r*min (Å)**	***r*max (Å)**
O^{2-} shell - Zr^{4+} shell	1274.1906	0.35729	10.551567	0	15
O^{2-} shell - O^{2-} shell	22764.3	0.149	24.407981	0	15
Zr^{4+} shell - Zr^{4+} shell	1.0	0.1	15.208590	0	15

(b) Shell model

$$E_{\text{sm}}^{\text{harmonic}} = \frac{1}{2}k_2(\delta r_i)^2$$

$$V_{ij}(r_{ij})^{\text{Coulomb}} = \frac{k_e q_i q_j}{r_{ij}}$$

**Species**	***Y*** **(e)**	***k*₂ (eV Å⁻²)**
Zr^{4+} shell	11.67	1157.7814
O^{2-} shell	-2.33	30.763136

Table S14. Shell-model interatomic potential derived for HfO_2.

(c) Buckingham potential

**Interaction**	***A*** **(eV)**	***ρ*** **(Å)**	***C*₆ (eV Å⁶)**	***r*min (Å)**	***r*max (Å)**
O^{2-} shell -Hf^{4+} shell	1393.3742	0.350844	13.002397	0	15
O^{2-} shell -O^{2-} shell	22764.3	0.149	18.169479	0	15
Hf^{4+} shell - Hf^{4+} shell	1.0	0.1	15.208590	0	15

(b) Shell model

**Species**	***Y*** **(e)**	***k*₂ (eV Å⁻²)**

Hf^{4+} shell	20.30	4304.6461
O^{2-} shell	-2.33	35.156403

Table S15. Bulk ionisation potential (eV) of CeO_2, ZrO_2 and HfO_2 predicted by the QM/MM approach using various hybrid DFT functionals.

Functional	CeO_2	c-ZrO_2	t-ZrO_2	m-ZrO_2	c-HfO_2	t-HfO_2	m-HfO_2
BB1K (42% HF)	5.38	5.84	6.03	6.08	5.49	5.73	5.77
PBE0 (25% HF)	5.10	5.45	5.78	5.77	5.05	5.42	5.39
HSE06 (25% HF)	4.99	5.33	5.65	5.67	4.91	5.26	5.32
B97-2 (21% HF)	4.92	5.25	5.59	5.60	4.83	5.18	5.22

Table S16. The bulk ionisation potential of CeO_2 predicted by the plane-wave DFT techniques using the "Core-level alignment" approach with (SM-CLA) and without (CLA) surface polarisation correction using shell-model (SM) potential.

	$\mathbf{IP_b^{CLA}}$ (eV)	$\mathbf{IP_b^{PSM-CLA}}$ (eV)
PBE+U ($U_{Ce\ 4f}$ = 5 eV)	4.61	3.58
PBE0	5.82	4.77
HSE06	5.45	4.40

Table S17. Comparison of several bulk and surface properties of group 2 rock-salt oxides, including gas-phase and in-lattice cation polarisability (α_M), in-lattice anion polarisability (α_O), static (ε_0) and high-frequency (ε_∞) dielectric constants, Madlung potential on the O site in bulk ($V_{\mathrm{Mad}}^{\mathrm{O,bulk}}$) calculated within 3D periodicity, bulk ionisation potential ($\mathrm{IP_b^{SM-CLA}}$), the magnitude of surface polarisation correction (D_s) determined by the SM-corrected CLA approach, surface ionisation potential ($\mathrm{IP_s^{(100)}}$). Some theoretical data calculated at the PBE0 level of theory were from Ref. [15, 121].

	MgO	CaO	SrO	BaO
Gas-phase α_M (a.u.)	0.478	3.266	5.824	10.417
In-lattice α_M (a.u.)	0.479	3.273	5.895	10.461
In-lattice α_O (a.u.)	6.319	6.347	6.962	6.597
ε_0[122]	9.9	11.95	13.1	34
ε_∞[15]	2.96	3.33	3.46	3.90
$V_{\mathrm{Mad}}^{\mathrm{O,bulk}}$ (V)	23.81	20.86	19.46	18.18
$\mathrm{IP_b^{SM-CLA}}$ (eV)[15]	6.89	5.64	5.21	4.14
D_s (eV)[15]	0.85	0.17	-0.06	-0.61

$IP_s^{(100)}$ (eV)[121]	6.52	5.26	4.73	4.18

Table S18. Ionisation potential of CeO_2 under different surface terminations predicted by plane-wave DFT calculations at different levels of theory, compared with experimental measurements by Wardenga and Klein[30].

	(100)O-t	(100)Ce-t	(100)CeO_4-t	(110)	(110)r	(111)
γ PBE+U (J m^{-2})	1.45	1.77	1.44	1.06	0.92	0.69
γ PBEsol+U (J m^{-2})	1.77	2.05	1.75	1.27	1.13	0.90
γ PBE0 (J m^{-2})	1.61	1.93	1.57	1.21	1.04	0.85
γ PBEsol0 (J m^{-2})	1.83			1.33		0.96
γ HSE06 (J m^{-2})	1.59			1.20		0.84
IP PBE+U (U=5 eV) (eV)	6.92	3.14	4.60	4.86	5.51	6.34
IP PBEsol+U (U=5 eV) (eV)	6.99	3.17	4.61	4.88	5.52	6.36
IP PBE0 (eV)	8.20	4.21	5.94	6.11	6.91	7.67
IP PBEsol0(eV)	8.29			6.13		7.71
IP HSE06(eV)	7.82			5.73		7.29
$V_{\mathrm{Mad}}^{\mathrm{O}}$ on relaxed surfaces (V)	24.94	19.67	22.78	22.70	23.30	23.99
IP Expt. (700 °C annealed in O_2)[30]	8.1			7.5		7.6
IP Expt. (700 °C annealed in Ar)[30]	7.1			6.5		6.5
IP Expt. (Treated with O plasma)[30]	9.1			9.0		8.6

Table S19. Shannon radii of cations,[123] calculated gas-phase cation polarisabilities (α_M) at the PBE0 level of theory, and the lowest Madelung potential on the O sites ($V_{\mathrm{Mad}}^{\mathrm{O}}$) in their constituted oxides. Apart from the original data from Walsh and Butler,[3] the cell parameters of CeO_2, HfO_2, ZrO_2, and TiO_2 were from DFT predictions at the PBE0 level of theory to be consistent with other analyses. Structures of new or updated entries were collected from the Cambridge Crystallographic Data Centre (CCDC) database with given reference numbers.

**Oxide**	**Space group Number**	**CCDC Number**	**Shannon radius of cation (Å)**	**α_M (a.u.)**	**Lowest $V_{\mathrm{Mad}}^{\mathrm{O}}$ (V)**
Li_2O	225		0.59	0.157	23.63
BeO	216		0.27	0.043	28.36
Na2O	225		0.99	0.825	19.63
MgO	225		0.72	0.478	22.17
Al_2O_3	167		0.535	0.269	26.36
SiO_2	122	1678590	0.26	0.164	30.54

K_2O	225		1.38	5.512	17.89
CaO	225		1	3.266	20.98
Sc_2O_3	206		0.745	2.127	23.63
TiO_2	136		0.605	1.475	26.32
Ti_2O_3	167		0.67	7.158	24.60
TiO	225		0.86	1.915	24.06
V_2O_5	59	1642259	0.46	1.757	25.17
V_2O_3	167		0.64	5.152	25.12
VO	225		0.79	1.075	24.73
CrO_2	136		0.55	1.180	26.55
Cr_2O_3	167		0.615	2.066	25.36
MnO_2	136		0.53	1.236	26.85
MnO	225		0.83	3.578	22.65
FeO	225		0.61	1.775	23.02
Fe_2O_3	167		0.645	2.902	24.88
CoO	225		0.745	2.396	23.68
NiO	166		0.69	2.352	24.13
Cu_2O	224		0.46	5.657	21.94
CuO	15		0.57	2.335	24.89
ZnO	186		0.6	2.286	23.88
Ga_2O_3	12		0.545	1.169	24.45
GeO_2	152		0.39	0.646	28.37
As_2O_5	92	1594900	0.3975	0.399	29.22
Rb_2O	225		1.52*	8.695	16.13
SrO	225		1.18	5.824	19.51
Y_2O_3	206	1702187	0.9	4.047	21.86
ZrO_2	14		0.78	2.946	22.90
ZrO_2	225		0.84	2.946	23.11
ZrO_2	137		0.84	2.946	23.30
Nb_2O_5	15		0.64	2.251	24.43
NbO_2	88		0.68	2.251	21.30
MoO_3	14	1706907	0.59	1.774	23.60
MoO_2	136		0.65	3.630	23.89
Tc_2O_7	61		0.37	1.433	25.92
RuO_4	15	1729382	0.36	1.181	29.13
RuO_2	136		0.62	3.635	25.60
Rh_2O_3	167		0.68	4.812	24.67
PdO	131		0.64	6.845	22.68
Pd_2O	224	1638049	0.59	10.593	21.79
Ag_2O	224		0.67	8.617	19.32
Ag_2O_3	224		0.75	4.198	22.28

CdO	225		0.95	4.901	21.44
In_2O_3	206		0.8	3.388	22.97
SnO_2	136		0.69	2.302	24.62
Sb_2O_5	15		0.6	1.693	23.08
TeO_2	96	1626060	0.66	7.814	23.08
Cs_2O	166		1.67*	15.823	14.08
BaO	225		1.35	10.417	18.03
La_2O_3	206	1759816	1.032	7.636	20.48
CeO_2	225		0.97	5.873	21.73
Ce_2O_3	164		1.07	7.633	20.08
Pr_2O_3	206	1762722	0.99	7.933	20.82
PrO_2	225	1691584	0.96	5.698	21.32
Nd_2O_3	206	1700456	0.983	7.393	20.92
Pm_2O_3	206	1762714	0.97	6.886	21.15
Sm_2O_3	206	1655575	0.958	6.480	21.13
Eu_2O_3	206	1703180	0.947	5.991	21.83
Gd_2O_3	206	1702175	0.938	5.838	21.28
Tb_2O_3	206	1762781	0.923	5.535	21.66
Dy_2O_3	206	1753704	0.912	5.335	21.85
Ho_2O_3	206	1693781	0.901	4.905	21.92
Er_2O_3	206	1609189	0.89	4.587	22.03
Tm_2O_3	206	1762818	0.88	4.510	22.21
YbO	225	1638098	1.02	6.361	20.64
Lu_2O_3	206	1702171	0.861	4.168	22.27
HfO_2	14		0.76	2.893	23.82
HfO_2	225		0.83	2.893	23.11
HfO_2	137		0.83	2.893	23.63
Ta_2O_5	59		0.64	2.329	23.16
WO_3	14	1615631	0.6	1.915	27.47
WO_2	136	1641007	0.66	4.557	23.82
Re_2O_7	19		0.53	1.603	25.62
OsO_4	15	1759474	0.39	1.382	27.98
IrO_2	136		0.625	4.600	25.39
PtO_2	58		0.625	4.709	25.23
Au_2O_3	43	1593820	0.68	6.297	22.41
HgO	62		1.02	7.594	20.88
Tl_2O	166		1.5*	20.729	14.13
Tl_2O_3	206		0.885	5.300	22.05
PbO_2	136		0.775	3.794	23.32
Bi_2O_3	14	1653670	1.03	9.916	20.01
PoO_2	225		1.08	7.512	20.80

Ac_2O_3	164	1605732	1.12*	9.950	18.11
ThO_2	225	1603819	1.05	7.680	20.95
PaO_2	225	1762700	1.01	6.871	21.28
UO_2	225	1604147	1	8.345	21.40
NpO_2	225	1762670	0.98	7.808	21.58
PuO_2	225	1619771	0.96	6.622	21.72

* Uncertain radius due to the lack of corresponding data with the required coordination number. Therefore, available values from the closest coordination number at the same charge state were selected.

3 Supporting Figure

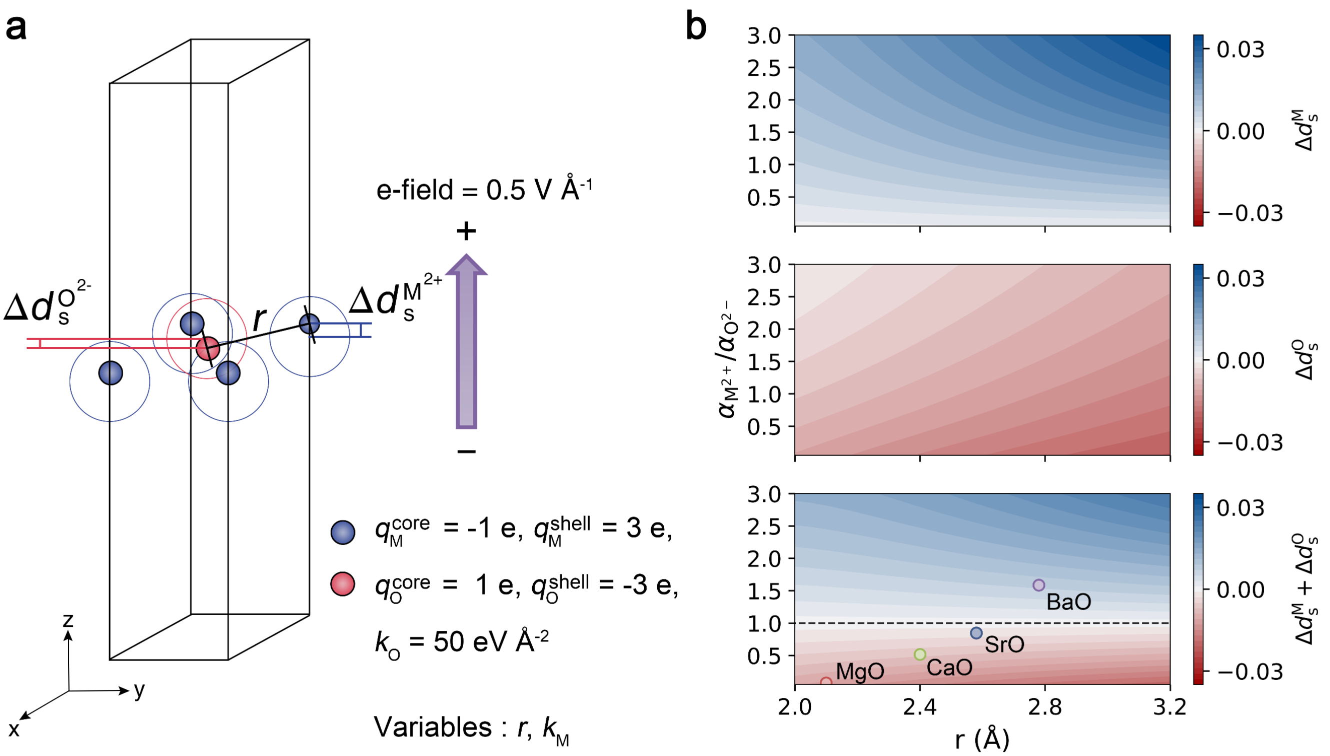

Figure S1. Polarisation of a rock-salt structured single-layer model in response to a constant electric field under the shell-model description. (a) Schematic diagram of the modelling system. In the simulations, the core and shell charges on both cation and anion sites, as well as the anion spring constant k_O are fixed. The M^{2+}-O^{2-} bond length r and cation spring constant k_M are varied to reproduce various lattice environments and cation polarisabilities ($\alpha_{M^{2+}} = (q_M^{shell})^2 / k_M$) in rock-salt structured oxides. (b) Variation of the cation (top), anion (middle) shell displacements, and their sum (bottom) under the electric field as a function of bond length and relative polarisability. The locations of MgO, CaO, SrO, and BaO are marked in circles in the bottom plot based on the calculated bond length and in-lattice ionic polarisability shown in Table S18. A clear sign transition in the sum of shell displacements is seen between SrO and BaO at $\alpha_{M^{2+}} / \alpha_{M^{2+}} = 1$, in which cation polarisability starts to prevail over anion polarisability, resulting in the transition in the direction of the surface dipole.

4 References

[1] N. F. Mott, R. W. Gurney, *Electronic processes in ionic crystals*, Clarendon Press, **1940**.

[2] E. Madelung, *Phys. Z* **1918**, *19*, 524-533.

[3] A. Walsh, K. T. Butler, *Acc. Chem. Res.* **2014**, *47*, 364-372.

[4] P. P. Ewald, *Annalen der physik* **1921**, *369*, 253-287.

[5] G. Lewis, C. Catlow, *J. Phys. C: Solid State Phys.* **1985**, *18*, 1149.

[6] a) N. Mott, M. Littleton, *Trans. Faraday Soc., 1940* **1939**, *35*, 1175; b) A. A. Sokol, S. A. French, S. T. Bromley, C. R. A. Catlow, H. J. van Dam, P. Sherwood, *Faraday Discuss.* **2007**, *134*, 267-282; c) Q. Hou, J. Buckeridge, T. Lazauskas, D. Mora-Fonz, A. A. Sokol, S. M. Woodley, C. R. A. Catlow, *J. Mater. Chem. C* **2018**, *6*, 12386-12395; d) X. Zhang, L. Zhu, Q. Hou, J. Guan, Y. Lu, T. W. Keal, J. Buckeridge, C. R. A. Catlow, A. A. Sokol, *Chem. Mater.* **2023**, *35*, 207-227.

[7] a) D. Mora-Fonz, J. Buckeridge, A. J. Logsdail, D. O. Scanlon, A. A. Sokol, S. Woodley, C. R. A. Catlow, *J. Phys. Chem. C* **2015**, *119*, 11598-11611; b) D. Mora-Fonz, T. Lazauskas, M. R. Farrow, C. R. A. Catlow, S. M. Woodley, A. A. Sokol, *Chem. Mater.* **2017**, *29*, 5306-5320.

[8] a) J. D. Gale, *J. Chem. Soc., Faraday Trans.* **1997**, *93*, 629-637; b) J. D. Gale, A. L. Rohl, *Mol. Simul.* **2003**, *29*, 291-341.

[9] M. L. GUPTA, S. Singh, *J. Am. Ceram. Soc.* **1970**, *53*, 663-665.

[10] D. H. Gay, A. L. Rohl, *J. Chem. Soc., Faraday Trans.* **1995**, *91*, 925-936.

[11] D. Parry, *Surf. Sci.* **1975**, *49*, 433-440.

[12] G. Kresse, J. Furthmüller, *Phys. Rev. B* **1996**, *54*, 11169.

[13] J. Ihm, A. Zunger, M. L. Cohen, *J. Phys. C: Solid State Phys.* **1979**, *12*, 4409.

[14] Y.-H. Li, A. Walsh, S. Chen, W.-J. Yin, J.-H. Yang, J. Li, J. L. Da Silva, X. Gong, S.-H. Wei, *Appl. Phys. Lett.* **2009**, *94*, 212109.

[15] A. J. Logsdail, D. O. Scanlon, C. R. A. Catlow, A. A. Sokol, *Phys. Rev. B* **2014**, *90*, 155106.

[16] P. E. Blöchl, *Phys. Rev. B* **1994**, *50*, 17953.

[17] C. Adamo, V. Barone, *J. Chem. Phys.* **1999**, *110*, 6158-6170.

[18] a) J. Heyd, G. E. Scuseria, M. Ernzerhof, *J. Chem. Phys.* **2003**, *118*, 8207-8215; b) A. V. Krukau, O. A. Vydrov, A. F. Izmaylov, G. E. Scuseria, *J. Chem. Phys.* **2006**, *125*.

[19] A. J. Garza, G. E. Scuseria, *J. Phys. Chem. Lett.* **2016**, *7*, 4165-4170.

[20] a) P. Sherwood, A. H. de Vries, M. F. Guest, G. Schreckenbach, C. R. A. Catlow, S. A. French, A. A. Sokol, S. T. Bromley, W. Thiel, A. J. Turner, *J. Mol. Struct.: THEOCHEM* **2003**, *632*, 1-28; b) A. A. Sokol, S. T. Bromley, S. A. French, C. R. A. Catlow, P. Sherwood, *Int. J. Quantum Chem.* **2004**, *99*, 695-712; c) D. O. Scanlon, C. W. Dunnill, J. Buckeridge, S. A. Shevlin, A. J. Logsdail, S. M. Woodley, C. R. A. Catlow, M. J. Powell, R. G. Palgrave, I. P. Parkin, *Nat. Mater.* **2013**, *12*, 798; d) C. R. A. Catlow, J. Buckeridge, M. R. Farrow, A. J. Logsdail, A. A. Sokol, in *Handbook of Solid State Chemistry, Vol. 5*, Wiley-VCH: Weinheim, Germany, **2017**, pp. 647-680.

[21] Y. Lu, M. R. Farrow, P. Fayon, A. J. Logsdail, A. A. Sokol, C. R. A. Catlow, P. Sherwood, T. W. Keal, *J. Chem. Theory Comput.* **2018**, *15*, 1317-1328.

[22] a) A. Walsh, J. Buckeridge, C. R. A. Catlow, A. J. Jackson, T. W. Keal, M. Miskufova, P. Sherwood, S. A. Shevlin, M. B. Watkins, S. M. Woodley, A. A. Sokol, *Chem. Mater.* **2013**, *25*,

2924-2926; b) J. Buckeridge, S. Bromley, A. Walsh, S. Woodley, C. Catlow, A. Sokol, *J. Chem. Phys.* **2013**, *139*, 124101; c) J. Buckeridge, K. T. Butler, C. R. A. Catlow, A. J. Logsdail, D. O. Scanlon, S. A. Shevlin, S. M. Woodley, A. A. Sokol, A. Walsh, *Chem. Mater.* **2015**, *27*, 3844-3851; d) A. J. Logsdail, C. A. Downing, T. W. Keal, P. Sherwood, A. A. Sokol, C. R. A. Catlow, *Phys. Chem. Chem. Phys.* **2016**, *18*, 28648-28660; e) J. Buckeridge, C. R. A. Catlow, M. Farrow, A. J. Logsdail, D. Scanlon, T. Keal, P. Sherwood, S. Woodley, A. Sokol, A. Walsh, *Phys. Rev. Mater.* **2018**, *2*, 054604.

[23] W. Jost, *J. Chem. Phys.* **1933**, *1*, 466-475.

[24] M. Valiev, E. J. Bylaska, N. Govind, K. Kowalski, T. P. Straatsma, H. J. Van Dam, D. Wang, J. Nieplocha, E. Apra, T. L. Windus, *Comput. Phys. Commun.* **2010**, *181*, 1477-1489.

[25] F. Weigend, R. Ahlrichs, *Phys. Chem. Chem. Phys.* **2005**, *7*, 3297-3305.

[26] D. Andrae, U. Haeussermann, M. Dolg, H. Stoll, H. Preuss, *Theor. Chim. Acta* **1990**, *77*, 123-141.

[27] Y. Zhao, B. J. Lynch, D. G. Truhlar, *J. Phys. Chem. A* **2004**, *108*, 2715-2719.

[28] P. J. Wilson, T. J. Bradley, D. J. Tozer, *J. Chem. Phys.* **2001**, *115*, 9233-9242.

[29] R. Gulde, P. Pollak, F. Weigend, *J. Chem. Theory Comput.* **2012**, *8*, 4062-4068.

[30] H. F. Wardenga, A. Klein, *Appl. Surf. Sci.* **2016**, *377*, 1-8.

[31] A. Pfau, K. Schierbaum, *Surf. Sci.* **1994**, *321*, 71-80.

[32] B. Wang, B. Zhu, S. Yun, W. Zhang, C. Xia, M. Afzal, Y. Cai, Y. Liu, Y. Wang, H. Wang, *NPG Asia Mater.* **2019**, *11*, 1-12.

[33] Y.-C. Huang, S.-H. Wu, C.-H. Hsiao, A.-T. Lee, M. H. Huang, *Chem. Mater.* **2020**, *32*, 2631-2638.

[34] X. Zheng, S. S. Mofarah, C. Cazorla, R. Daiyan, A. A. Esmailpour, J. Scott, Y. Yao, S. Lim, V. Wong, E. Y. Chen, *Adv. Funct. Mater.* **2021**, *31*, 2103171.

[35] F.-C. Chiu, C.-M. Lai, *J. Phys. D: Appl. Phys.* **2010**, *43*, 075104.

[36] W.-c. Shih, C.-H. Chen, F.-C. Chiu, C.-M. Lai, H.-L. Hwang, *ECS Trans.* **2010**, *28*, 435.

[37] J. C. Wang, K. C. Chiang, T. F. Lei, C. L. Lee, *Electrochem. Solid-State Lett.* **2004**, *7*, E55.

[38] F.-C. Chiu, *Electrochem. Solid-State Lett.* **2008**, *11*, H135.

[39] H. B. Michaelson, *J. Appl. Phys.* **1977**, *48*, 4729-4733.

[40] N. K. R. Nallabala, S. Godavarthi, V. K. Kummara, M. K. Kesarla, D. Saha, H. S. Akkera, G. K. Guntupalli, S. Kumar, S. P. Vattikuti, *Mater. Sci. Semicond. Process.* **2020**, *117*, 105190.

[41] S.-M. Yang, C.-H. Chien, J.-J. Huang, T.-F. Lei, M.-J. Tsai, L.-S. Lee, *Appl. Phys. Lett.* **2007**, *91*, 262104.

[42] N. Fujimura, A. Ohta, K. Makihara, S. Miyazaki, *Jpn. J. Appl. Phys.* **2016**, *55*, 08PC06.

[43] Y. Zhu, N. Jain, M. K. Hudait, D. Maurya, R. Varghese, S. Priya, *J. Vac. Sci. Technol., B: Nanotechnol. Microelectron.: Mater., Process., Meas., Phenom.* **2014**, *32*, 011217.

[44] Y.-T. Cheng, Y.-H. Lin, W.-S. Chen, K.-Y. Lin, H.-W. Wan, C.-P. Cheng, H.-H. Cheng, J. Kwo, M. Hong, T.-W. Pi, *Appl. Phys. Express* **2017**, *10*, 075701.

[45] X.-J. Wen, C.-G. Niu, L. Zhang, C. Liang, G.-M. Zeng, *Appl. Catal., B* **2018**, *221*, 701-714.

[46] Y. Hinuma, T. Toyao, T. Kamachi, Z. Maeno, S. Takakusagi, S. Furukawa, I. Takigawa, K.-i. Shimizu, *J. Phys. Chem. C* **2018**, *122*, 29435-29444.

[47] Q. Qiao, K. Yang, L.-L. Ma, W.-Q. Huang, B.-X. Zhou, A. Pan, W. Hu, X. Fan, G.-F. Huang, *J. Phys. D: Appl. Phys.* **2018**, *51*, 275302.
[48] E. Cerrato, C. Gionco, M. C. Paganini, E. Giamello, E. Albanese, G. Pacchioni, *ACS Appl. Energy Mater.* **2018**, *1*, 4247-4260.
[49] a) G. Darbandy, R. Ritzenthaler, F. Lime, I. Garduno, M. Estrada, A. Cerdeira, B. Iniguez, *Semicond. Sci. Technol.* **2011**, *26*, 045002; b) O. Engström, B. Raeissi, S. Hall, O. Buiu, M. C. Lemme, H. Gottlob, P. Hurley, K. Cherkaoui, *Solid-State Electron.* **2007**, *51*, 622-626.
[50] V. Afanas' ev, A. Stesmans, *Appl. Phys. Lett.* **2004**, *84*, 2319-2321.
[51] S. A. Corrêa, S. Brizzi, D. Schmeisser, *J. Vac. Sci. Technol., A* **2016**, *34*, 01A117.
[52] Z. Liu, W. Chim, S. Chiam, J. Pan, C. Ng, *J. Mater. Chem.* **2012**, *22*, 17887-17892.
[53] V. V. Afanas' ev, *Advances in Condensed Matter Physics* **2014**, *2014*.
[54] H. C. Shin, L. S. Son, K. R. Kim, S. K. Oh, H. J. Kang, D. Tahir, S. Heo, J. G. Chung, J. C. Lee, S. Tougaard, *J. Surf. Anal.* **2011**, *17*, 203-207.
[55] H.-S. Jung, J. H. Jang, D.-Y. Cho, S.-H. Jeon, H. K. Kim, S. Y. Lee, C. S. Hwang, *Electrochem. Solid-State Lett.* **2011**, *14*, G17.
[56] V. Afanas' ev, S. Shamuilia, A. Stesmans, A. Dimoulas, Y. Panayiotatos, A. Sotiropoulos, M. Houssa, D. Brunco, *Appl. Phys. Lett.* **2006**, *88*, 132111.
[57] V. Fiorentini, G. Gulleri, *Phys. Rev. Lett.* **2002**, *89*, 266101.
[58] J. Robertson, *J. Vac. Sci. Technol., B: Microelectron. Nanometer Struct.--Process., Meas., Phenom.* **2000**, *18*, 1785-1791.
[59] Z. Zhang, Y. Guo, H. Lu, S. J. Clark, J. Robertson, *Appl. Phys. Lett.* **2020**, *116*, 131602.
[60] J. Lyons, A. Janotti, C. Van de Walle, *Microelectron. Eng.* **2011**, *88*, 1452-1456.
[61] H.-D. Wiemhöfer, S. Harke, U. Vohrer, *Solid State Ionics* **1990**, *40*, 433-439.
[62] L. Chai, R. White, M. T. Greiner, Z.-H. Lu, *Phys. Rev. B* **2014**, *89*, 035202.
[63] H. Nohira, W. Tsai, W. Besling, E. Young, J. Pétry, T. Conard, W. Vandervorst, S. De Gendt, M. Heyns, J. Maes, *J. Non-Cryst. Solids* **2002**, *303*, 83-87.
[64] L. Zhu, Q. Fang, G. He, M. Liu, L. Zhang, *J. Phys. D: Appl. Phys.* **2006**, *39*, 5285.
[65] S. Miyazaki, *J. Vac. Sci. Technol., B: Microelectron. Nanometer Struct.--Process., Meas., Phenom.* **2001**, *19*, 2212-2216.
[66] a) P. Aldebert, J. P. TRAVERSE, *J. Am. Ceram. Soc.* **1985**, *68*, 34-40; b) E. Stefanovich, A. L. Shluger, C. Catlow, *Phys. Rev. B* **1994**, *49*, 11560.
[67] R. French, S. Glass, F. Ohuchi, Y.-N. Xu, W. Ching, *Phys. Rev. B* **1994**, *49*, 5133.
[68] M. T. Lanagan, J. K. Yamamoto, A. Bhalla, S. Sankar, *Mater. Lett.* **1989**, *7*, 437-440.
[69] A. Dwivedi, A. N. Cormack, *Philos. Mag. A* **1990**, *61*, 1-22.
[70] D. L. Wood, K. Nassau, *Appl. Opt.* **1982**, *21*, 2978-2981.
[71] J. Adam, M. Rogers, *Acta Crystallogr.* **1959**, *12*, 951-951.
[72] A. Feinberg, C. Perry, *J. Phys. Chem. Solids* **1981**, *42*, 513-518.
[73] J. Zhu, Z. Liu, *Appl. Phys. A* **2004**, *78*, 741-744.
[74] I. El-Shanshoury, V. Rudenko, I. Ibrahim, *J. Am. Ceram. Soc.* **1970**, *53*, 264-268.
[75] S.-G. Lim, S. Kriventsov, T. N. Jackson, J. Haeni, D. G. Schlom, A. Balbashov, R. Uecker, P. Reiche, J. Freeouf, G. Lucovsky, *J. Appl. Phys.* **2002**, *91*, 4500-4505.

[76] A. Toriumi, *ECS Trans.* **2017**, *80*, 29.

[77] D. L. Wood, K. Nassau, T. Kometani, D. Nash, *Appl. Opt.* **1990**, *29*, 604-607.

[78] Z. Qiu, C.-H. Li, J. R. Jinschek, P.-I. Gouma, *Ceram. Int.* **2021**, *47*, 14208-14215.

[79] C.-H. Fu, K.-S. Chang-Liao, C.-C. Li, Z.-H. Ye, F.-M. Hsu, T.-K. Wang, Y.-J. Lee, M.-J. Tsai, *Appl. Phys. Lett.* **2012**, *101*, 032105.

[80] Y.-S. Kang, D.-K. Kim, H.-K. Kang, S. Cho, S. Choi, H. Kim, J.-H. Seo, J. Lee, M.-H. Cho, *J. Phys. Chem. C* **2015**, *119*, 6001-6008.

[81] T. Boscke, S. Govindarajan, C. Fachmann, J. Heitmann, A. Avellan, U. Schroder, S. Kudelka, P. Kirsch, C. Krug, P. Hung, in *2006 International Electron Devices Meeting*, IEEE, **2006**, pp. 1-4.

[82] M. Villanueva-Ibanez, C. Le Luyer, S. Parola, O. Marty, J. Mugnier, *Rev. Adv. Mater. Sci.* **2003**, *5*, 296-301.

[83] Z. Mei-Qiong, Z. Dong-Ping, T. Tian-Ya, H. Hong-Bo, S. Jian-Da, F. Zheng-Xiu, *Chin. Phys. Lett.* **2005**, *22*, 1246.

[84] M. Balog, M. Schieber, M. Michman, S. Patai, *Thin Solid Films* **1977**, *41*, 247-259.

[85] K. Kukli, J. Ihanus, M. Ritala, M. Leskela, *Appl. Phys. Lett.* **1996**, *68*, 3737-3739.

[86] E. Meagher, G. A. Lager, *Can. Mineral.* **1979**, *17*, 77-85.

[87] A. Amtout, R. Leonelli, *Phys. Rev. B* **1995**, *51*, 6842.

[88] R. A. Parker, *Phys. Rev.* **1961**, *124*, 1719.

[89] G. Samara, P. Peercy, *Phys. Rev. B* **1973**, *7*, 1131.

[90] J. G. Traylor, H. Smith, R. Nicklow, M. Wilkinson, *Phys. Rev. B* **1971**, *3*, 3457.

[91] S. Wemple, *J. Chem. Phys.* **1977**, *67*, 2151-2168.

[92] P. J. Bhatt, L. J. Tomar, R. K. Desai, B. S. Chakrabarty, in *AIP Conf. Proc., Vol. 1665*, AIP Publishing LLC, **2015**, p. 050125.

[93] M. Horn, C. Schwebdtfeger, E. Meagher, *Z. Kristallogr. Cryst. Mater.* **1972**, *136*, 273-281.

[94] B. Kraeutler, A. J. Bard, *J. Am. Chem. Soc.* **1978**, *100*, 5985-5992.

[95] R. Gonzalez, R. Zallen, H. Berger, *Phys. Rev. B* **1997**, *55*, 7014.

[96] L. Berberich, M. Bell, *J. Appl. Phys.* **1940**, *11*, 681-692.

[97] J. Y. Kim, H. S. Jung, J. H. No, J.-R. Kim, K. S. Hong, *J. Electroceram.* **2006**, *16*, 447-451.

[98] A. Di Paola, M. Bellardita, L. Palmisano, *Catalysts* **2013**, *3*, 36-73.

[99] W. Hu, L. Li, G. Li, C. Tang, L. Sun, *Cryst. Growth Des.* **2009**, *9*, 3676-3682.

[100] S. M. Qaid, M. Hussain, M. Hezam, M. M. Khan, H. Albrithen, H. M. Ghaithan, A. S. Aldwayyan, *Mater. Chem. Phys.* **2019**, *225*, 55-59.

[101] M. Capdevila-Cortada, N. López, *Nat. Mater.* **2017**, *16*, 328-334.

[102] L. Brugnoli, M. C. Menziani, S. Urata, A. Pedone, *J. Phys. Chem. A* **2021**, *125*, 5693-5708.

[103] a) H. Nörenberg, J. Harding, *Surf. Sci.* **2001**, *477*, 17-24; b) C. Yang, X. Yu, S. Heißler, A. Nefedov, S. Colussi, J. Llorca, A. Trovarelli, Y. Wang, C. Wöll, *Angew. Chem., Int. Ed.* **2017**, *56*, 375-379.

[104] D. C. Grinter, M. Allan, H. J. Yang, A. Salcedo, G. E. Murgida, B.-J. Shaw, C. L. Pang, H. Idriss, M. V. Ganduglia-Pirovano, G. Thornton, *Angew. Chem., Int. Ed.* **2021**, *60*, 13835-13839.

[105] O. Matz, M. Calatayud, *ACS Omega* **2018**, *3*, 16063-16073.

[106] Y.-L. Yang, X.-L. Fan, C. Liu, R.-X. Ran, *Phys. B* **2014**, *434*, 7-13.

[107] M. J. Wolf, K. P. McKenna, A. L. Shluger, *J. Phys. Chem. C* **2012**, *116*, 25888-25897.

[108] W. Piskorz, J. Grybos, F. Zasada, P. Zapała, S. Cristol, J.-F. o. Paul, Z. Sojka, *J. Phys. Chem. C* **2012**, *116*, 19307-19320.

[109] W. Piskorz, J. Grybos, F. Zasada, S. Cristol, J.-F. Paul, A. Adamski, Z. Sojka, *J. Phys. Chem. C* **2011**, *115*, 24274-24286.

[110] A. B. Mukhopadhyay, J. F. Sanz, C. B. Musgrave, *Phys. Rev. B* **2006**, *73*, 115330.

[111] R. Batra, H. D. Tran, R. Ramprasad, *Appl. Phys. Lett.* **2016**, *108*, 172902.

[112] H. Perron, C. Domain, J. Roques, R. Drot, E. Simoni, H. Catalette, *Theor. Chem. Acc.* **2007**, *117*, 565-574.

[113] A. Beltrán, J. Andres, J. R. Sambrano, E. Longo, *J. Phys. Chem. A* **2008**, *112*, 8943-8952.

[114] M. Lazzeri, A. Vittadini, A. Selloni, *Phys. Rev. B* **2001**, *63*, 155409.

[115] F. Labat, P. Baranek, C. Adamo, *J. Chem. Theory Comput.* **2008**, *4*, 341-352.

[116] A. Beltran, L. Gracia, J. Andres, *J. Phys. Chem. B* **2006**, *110*, 23417-23423.

[117] T. R. Esch, I. Gadaczek, T. Bredow, *Appl. Surf. Sci.* **2014**, *288*, 275-287.

[118] X.-Q. Gong, A. Selloni, *Phys. Rev. B* **2007**, *76*, 235307.

[119] M. Dolg, H. Stoll, H. Preuss, *J. Chem. Phys.* **1989**, *90*, 1730-1734.

[120] J. K. Desmarais, A. Erba, R. Dovesi, *Theor. Chem. Acc.* **2018**, *137*, 1-11.

[121] A. J. Logsdail, D. Mora-Fonz, D. O. Scanlon, C. R. A. Catlow, A. A. Sokol, *Surf. Sci.* **2015**, *642*, 58-65.

[122] a) M. Subramanian, R. Shannon, B. Chai, M. Abraham, M. Wintersgill, *Phys. Chem. Miner.* **1989**, *16*, 741-746; b) R. S. Bever, R. L. Sproull, *Phys. Rev.* **1951**, *83*, 801; c) J. Jacobson, E. Nixon, *J. Phys. Chem. Solids* **1968**, *29*, 967-976.

[123] R. D. Shannon, *Acta Crystallogr., Sect. A: Cryst. Phys., Diffr., Theor. Gen. Crystallogr.* **1976**, *32*, 751-767.
